# Efficient homing of antibody-secreting cells to the bone marrow requires RNA-binding protein ZFP36L1

**DOI:** 10.1084/jem.20200504

**Published:** 2020-12-11

**Authors:** Alexander Saveliev, Sarah E. Bell, Martin Turner

**Affiliations:** Laboratory of Lymphocyte Signalling and Development, The Babraham Institute, Cambridge, UK

## Abstract

Cell migration relies on coordinated activity of chemotactic and guidance receptors. Here, we report a specific role for the RNA-binding protein ZFP36L1 in limiting the abundance of molecules involved in the homing of antibody-secreting cells (ASCs) to the bone marrow (BM). In the absence of ZFP36L1, ASCs build up in the spleen and the liver and show diminished accumulation in the BM. ZFP36L1 facilitates migration by directly regulating G protein–coupled receptor kinase 2 (GRK2) and the integrin chains α4 and β1 in splenic ASCs. Expression of CXCR4 and of the integrins α4 and β1 is differentially regulated on ASCs produced at the early and late stages of the immune response. Consequently, deletion of the *Zfp36l1* gene has a stronger effect on BM accumulation of high-affinity ASCs formed late in the response. Thus, ZFP36L1 is an integral part of the regulatory network controlling gene expression during ASC homing.

## Teaser

Splenic ASCs generated late in the immune response display increased surface abundance of CXCR4 and of the integrins α4 and β1. ZFP36L1 controls the range of integrin expression, facilitating transition of ASCs to their survival niche in the bone marrow.

## Introduction

Long-term humoral immunity is derived from the generation and persistence of memory B cells and antibody-secreting cells (ASCs) following infection. It is generally accepted that long-lived ASCs are formed in secondary lymphoid organs from B cells after they undergo affinity maturation of Igs in germinal centers (GCs; Victora and Nussenzweig, 2012; Suan et al., 2017; Nutt et al., 2015; Shlomchik and Weisel, 2012). Newly generated ASCs then migrate to the bone marrow (BM), where ASC survival and function are sustained for extended periods of time (Slifka et al., 1998; Manz et al., 1997). Understanding the mechanisms regulating ASC homing is thus important for improving vaccine efficacy and immunity.

Egress of ASCs from the spleen depends on the action of the chemokine CXCL12 and its receptor CXCR4, as well as sphingosine-1-phosphate (S1P) and its receptor S1PR1 (Hargreaves et al., 2001; Kabashima et al., 2006; Lu and Cyster, 2019). Once in the blood, ASC homing to the BM is guided primarily by the CXCL12/CXCR4 pair (Hauser et al., 2002; Bowman et al., 2002; Luther et al., 2002). The integrin dimer α4β1 activated by CXCR4 signaling mediates rolling, firm adhesion, and arrest on the fenestrated endothelium lining BM sinusoids (Chan et al., 2001; Peled et al., 2000; Grabovsky et al., 2000). Recently, it was shown that reduced activation of the integrin β1 on early ASCs in mice deficient for the cochaperone Mzb1 was associated with their impaired trafficking to the BM (Andreani et al., 2018). While another integrin dimer, α4β7, is mainly thought of as an adhesion molecule directing migration of lymphocytes to the intestine, antibody-blocking and genetic experiments also suggest a role for this integrin in BM homing (Katayama et al., 2004; Murakami et al., 2016).

It is known that the adhesive properties of integrins must be precisely regulated (Bouvard et al., 2013) and that excessive surface abundance of integrins, or their abnormal activation, can inhibit rather than promote chemokine-induced migration (Imai et al., 2008; Lu and Cyster, 2002). In this way, the defective accumulation of ASCs lacking the tyrosine phosphatase SHP1 (encoded by *Ptpn6*) in the BM of mice has been linked to aberrant activation of the integrin α4β1 (Li et al., 2014), while impaired de-adhesion of α4β7 has been reported to affect migration of lymphocytes to the gut (Park et al., 2007). Dynamic modulation of S1PR1 and CXCR4 activity is also known to be critical for the migration and survival of ASCs (Arnon et al., 2011; Biajoux et al., 2016). While signaling pathways mediate acute responses to adhesive and chemotactic cues, transcription factor–mediated control of mRNA abundance is thought to be the principal mechanism by which ASCs reprogram their adhesive and chemotactic properties.

Transcription factors such as BLIMP1, c-Myb, and KLF2 couple differentiation to adhesion and migration (Minnich et al., 2016; Winkelmann et al., 2011; Good-Jacobson et al., 2015). However, the rapid and transient repression of gene expression or the localized translation that would enable complex cell behavior is not well suited to transcriptional regulation. Hence, posttranscriptional control of gene expression is increasingly recognized as having an essential contribution to the differentiation, migration, and longevity of ASCs (Corcoran and Tarlinton, 2016; Khodadadi et al., 2019). MicroRNA-mediated regulation appears to be a common feature of these processes (Porstner et al., 2015; Lu et al., 2014; Vigorito et al., 2007; Basso et al., 2012). In particular, mir-17-92 has been shown to directly control ASC homing to the BM by targeting the 3′ untranslated region (3′ UTR) of S1PR1 mRNA (Xu et al., 2015). In contrast, roles for RNA-binding proteins in ASC trafficking and survival have yet to be identified.

ZFP36L1 is a member of the ZFP36 family of RNA-binding proteins that can interact with AU-rich elements in the 3′ UTR of mRNA and attenuate expression of the corresponding genes (Brooks and Blackshear, 2013). In lymphocytes, it has been shown to play redundant roles with its paralogue ZFP36L2 in developing T and B cells (Hodson et al., 2010; Galloway et al., 2016; Vogel et al., 2016). More recently, ZFP36L1 was demonstrated to have an indispensable function in maintaining the population of marginal zone (MZ) B cells (Newman et al., 2017). The roles of ZFP36L1 in the GC response and immunological memory are unknown. Here, we report a specific requirement for ZFP36L1 in the BM homing of ASCs. By limiting the expression of key molecules mediating migration, ZFP36L1 facilitates unimpeded transition of ASCs from secondary lymphoid organs to their survival niche in the BM, thereby promoting the establishment of long-term ASCs.

## Results

### ZFP36L1 in B cells determines the numbers of BM ASCs

**Figure S1.**
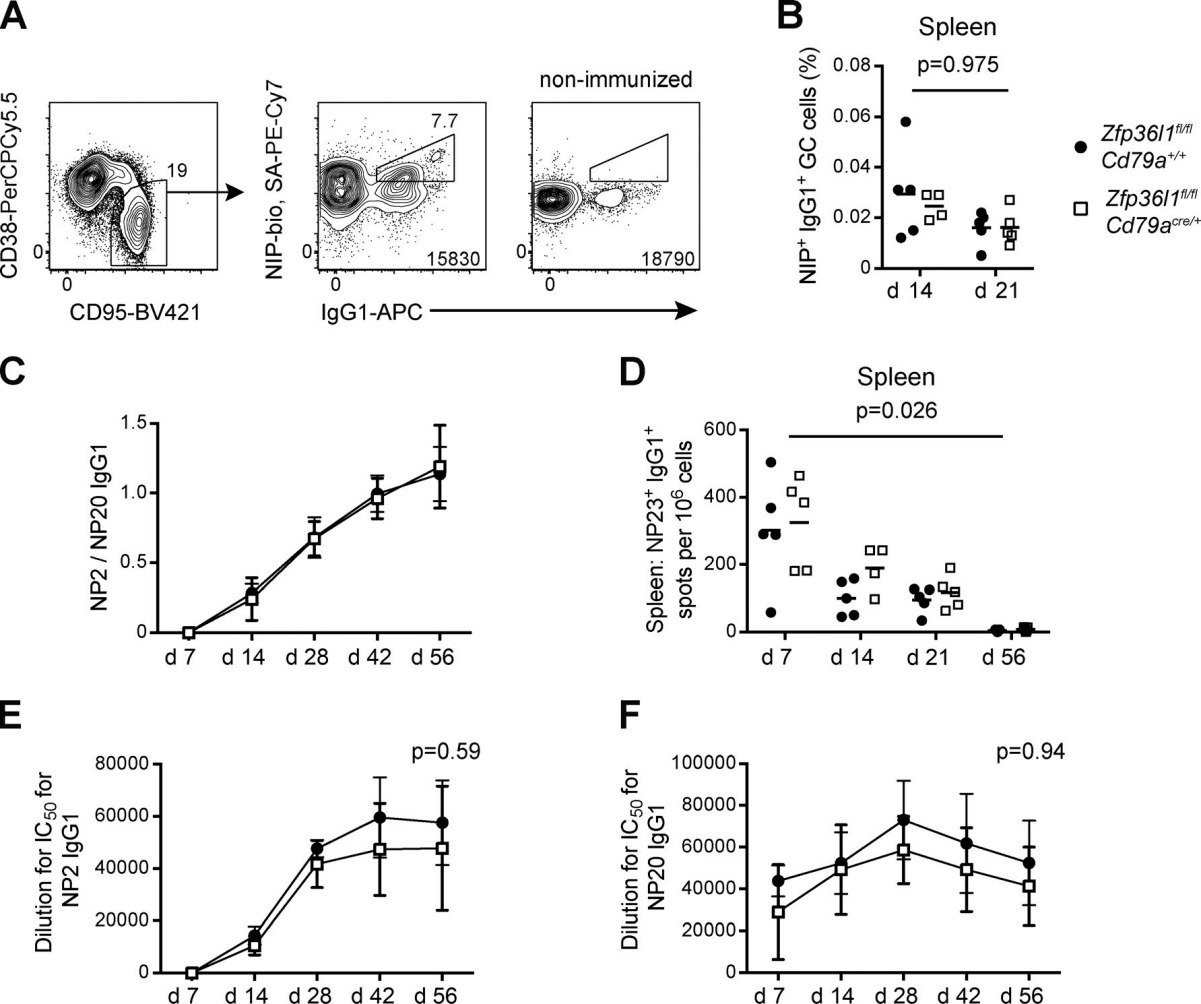
**The GC reaction is not affected in the absence of ZFP36L1 in B cells.** **(A)** Representative flow cytometry plots used for detection of NP-specific GC B cells; surface NIP^+^, IgG1^+^ cells (middle plot) were identified in the population of CD95^+^CD38^low^ GC cells (left plot, pregated on eFluor780^−^IgM^−^IgD^−^CD90.2^−^ cells), and the absence of such NIP^+^, IgG1^+^ cells was confirmed in a nonimmunized mouse (right plot). **(B)** Frequency of surface NIP^+^IgG1^+^ GC cells in the spleens *of Zfp36l1^fl/fl^ CD79a^+/+^* (closed circles) and *Zfp36l1^fl/fl^ CD79a^Cre/+^* (open squares) mice at days 14 and 21 after immunization; the number of surface NIP^+^IgG1^+^ GC cells in the spleen is normalized to the number of all live CD19^+^ cells (*n* ≥ 4; two-way ANOVA on logarithmically transformed data; the P value shown is for the main genotype effect). For all plots in all panels, each symbol represents data from an individual mouse, and lines represent means. Data are representative of two independent experiments. **(C)** Ratio of anti-NP_2_ IgG1 to anti-NP_20_ IgG1 (NP_2_/NP_20_) antibody in the serum of *Zfp36l1^fl/fl^ CD79a^+/+^* (closed circles) and *Zfp36l1^fl/fl^ CD79a^Cre/+^* (open squares) mice measured by ELISA at various time points after immunization with NP-KLH in alum. **(D)** ELISPOT analysis of NP-specific ASCs in *Zfp36l1^fl/fl^ CD79a^+/+^* and *Zfp36l1^fl/fl^ CD79a^Cre/+^* mice. Enumeration of ASCs secreting anti-NP_23_ (high and low affinity) IgG1 antibody in the spleen (*n* ≥ 4; two-way ANOVA on logarithmically transformed data; the P value shown is for the main genotype effect). **(E and F)** Titers of anti-NP_2_ IgG1 (E) and anti-NP_20_ IgG1 (F) antibody in the serum of *Zfp36l1^fl/fl^ CD79a^+/+^* (closed circles) and *Zfp36l1^fl/fl^ CD79a^Cre/+^* (open squares) mice (each data point is a mean ± SD of dilution for a 50% inhibitory concentration for *n* = 5 mice for each genotype; two-way ANOVA on logarithmically transformed data with Sidak’s correction for multiple testing; the P value shown is for the genotype effect for day 56). For all flow cytometry plots, the numbers next to the enclosed areas (gates) represent percentages of the cells falling into the gate, and the number in the bottom right corner shows the number of cells in the plot. SA, streptavidin; PE, R-phycoerythrin.

**Figure 1.**
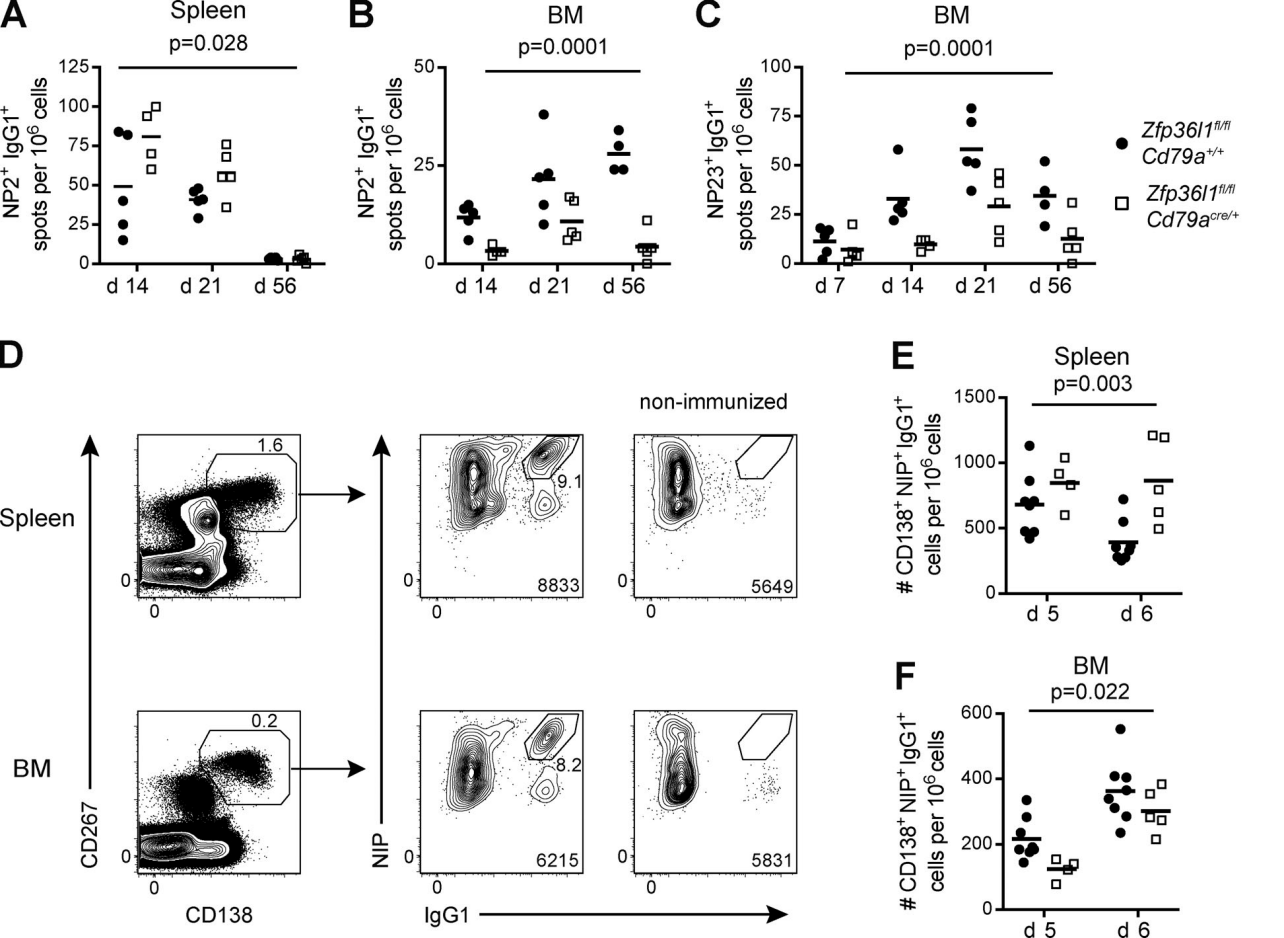
**Accumulation of Ag-specific ASCs in the BM but not their generation in the spleen is impaired after both primary immunization and reimmunization in *Zfp36l1^fl/fl^ CD79a^Cre/+^* mice.** **(A–C)** ELISPOT analysis of NP-specific ASCs in *Zfp36l1^fl/fl^ CD79a^+/+^* (closed circles) and *Zfp36l1^fl/fl^ CD79a^Cre/+^* (open squares) mice. Enumeration of ASCs secreting anti-NP_2_ (high affinity) IgG1 antibody in the spleen (A) and BM (B) and of ASCs secreting anti-NP_23_ (high and low affinity) IgG1 antibody in the BM (C). For all plots in all panels, each symbol displays data from an individual mouse, lines represent means, and data are representative of two independent experiments for day 14 (*n* ≥ 4; two-way ANOVA on logarithmically transformed data; the P values shown are for the main genotype effect). **(D)** Representative flow cytometry plots showing intracellular staining with IgG1 antibody and NIP-BSA-biotin to identify NP-specific ASCs in the spleen (three top plots) and the BM (three bottom plots); intracellular NIP^hi^, IgG1^hi^ cells (middle plots) were identified in the population of all CD138^+^CD267^+^ cells (left plots, gated on eFluor780^−^CD3^−^IgD^−^ cells), and their absence was confirmed in a nonimmunized mouse (right plots). **(E and F)** Quantification of CD138^+^NIP^+^IgG1^+^ cells in *Zfp36l1^fl/fl^ CD79a^+/+^* (closed circles) and *Zfp36l1^fl/fl^ CD79a^Cre/+^* (open squares) mice at days 5 and 6 after reimmunization in the spleen (E) and the BM (F) normalized to the numbers of all eFluor780^−^ cells in the sample (*n* ≥ 4; two-way ANOVA; the P values shown are for the main genotype effect). For all flow cytometry plots, the numbers next to the enclosed areas (gates) represent percentages of the cells falling into the gate, and the number in the bottom right corner shows the number of cells in the plot.

To identify a role for ZFP36L1 in B cells during humoral immune responses, we used mice with B cell–specific deletion of a conditional *Zfp36l1* allele (*Zfp36l1*^*fl*^; Newman et al., 2017). At 14 and 21 d following immunization with alum-precipitated 4-hydroxy-5-nitrophenylacetyl (NP) conjugated to KLH, *Zfp36l1^fl/fl^ CD79a^+/+^* (control) and *Zfp36l1^fl/fl^ CD79a^Cre/+^* (Zfp36l1 conditional KO [Zfp36l1 cKO]) mice had similar numbers of NP-binding IgG1^+^ GC B cells in the spleen (Fig. S1, A and B). The affinity maturation of NP-reactive IgG1 antibody, as determined by the ratio of serum antibody with high affinity to antibody with all affinities, was evident early in the immune response and indistinguishable between Zfp36l1 cKO and control mice (Fig. S1 C). Furthermore, the number of NP-2–binding (high affinity) IgG1-secreting ASCs, as enumerated by ELISPOT, was slightly increased in the spleens of the *Zfp36l1^fl/fl^ CD79a^Cre/+^* mice compared with that of *Zfp36l1^fl/fl^ CD79a^+/+^* mice (Fig. 1 A). This was also true for ASCs secreting NP-reactive antibody irrespective of affinity (Fig. S1 D). Thus, the GC response in the spleen shows no impairment when ZFP36L1 is absent from B cells. Despite this, the frequency of NP-specific ASCs in the BM of *Zfp36l1^fl/fl^ CD79a^Cre/+^* mice did not reach the level observed in *Zfp36l1^fl/fl^ CD79a^+/+^* mice (Fig. 1, B and C), resulting in a modest decrease of NP-specific antibody in serum (Fig. S1, E and F). Failure by serum antibody titers to closely follow the reduction in the ASC numbers is not unprecedented (Takahashi et al., 1998; Holl et al., 2011) and probably reflects the high productivity of ASCs (Hibi and Dosch, 1986; Taubenheim et al., 2012) and variability in Ig production by individual cells. In summary, while the initial stages of ASC differentiation in the spleen take place independently of ZFP36L1, it is required for their accumulation in the BM. This could reflect a role for ZFP36L1 in the migration of ASCs to the BM and in their subsequent survival.

The protracted nature of a primary immune response, the paucity of ASCs generated, and the diminished expression of surface Ig upon ASC maturation prevents easy identification and tracking of newly formed antigen-specific ASCs. Therefore, to address questions about the role of ZFP36L1 in ASCs, we set up a system that allowed us to follow a synchronized wave of NP-specific ASCs. We reimmunized mice with NP-KLH in alum 21 d after primary immunization and used intracellular staining for IgG1 antibody recognizing 4-hydroxy-3-iodo-5-nitrophenylacetic acid (NIP) to identify elicited ASCs among all eFluor780^−^IgD^−^CD4^−^CD8^−^CD138^+^CD267^+^ cells (Fig. 1 D). The absence of NIP^hi^IgG1^hi^ cells (subsequently referred to as CD138^+^NIP^+^IgG1^+^ cells) in nonimmunized mice confirmed the specificity of staining (Fig. 1 D,****rightmost plots), while their low frequency (8–9% at day 5 after reimmunization) among all CD138^+^CD267^+^ cells (Fig. 1 D, middle plots) underscored the limitations of analyzing the whole CD138^+^CD267^+^ population when following the fate of elicited ASCs. Enumeration of CD138^+^NIP^+^IgG1^+^ cells in the spleen and BM of *Zfp36l1^fl/fl^ CD79a^Cre/+^* and *Zfp36l1^fl/fl^ CD79a^+/+^* mice after reimmunization (Fig. 1, E and F) recapitulated the phenotype observed in the primary response. The consistency of observations in both settings indicates that reimmunization can be useful for understanding specific defects in ZFP36L1-deficient ASCs.

### ZFP36L1 controls migration of newly formed ASCs to the BM

**Figure 2.**
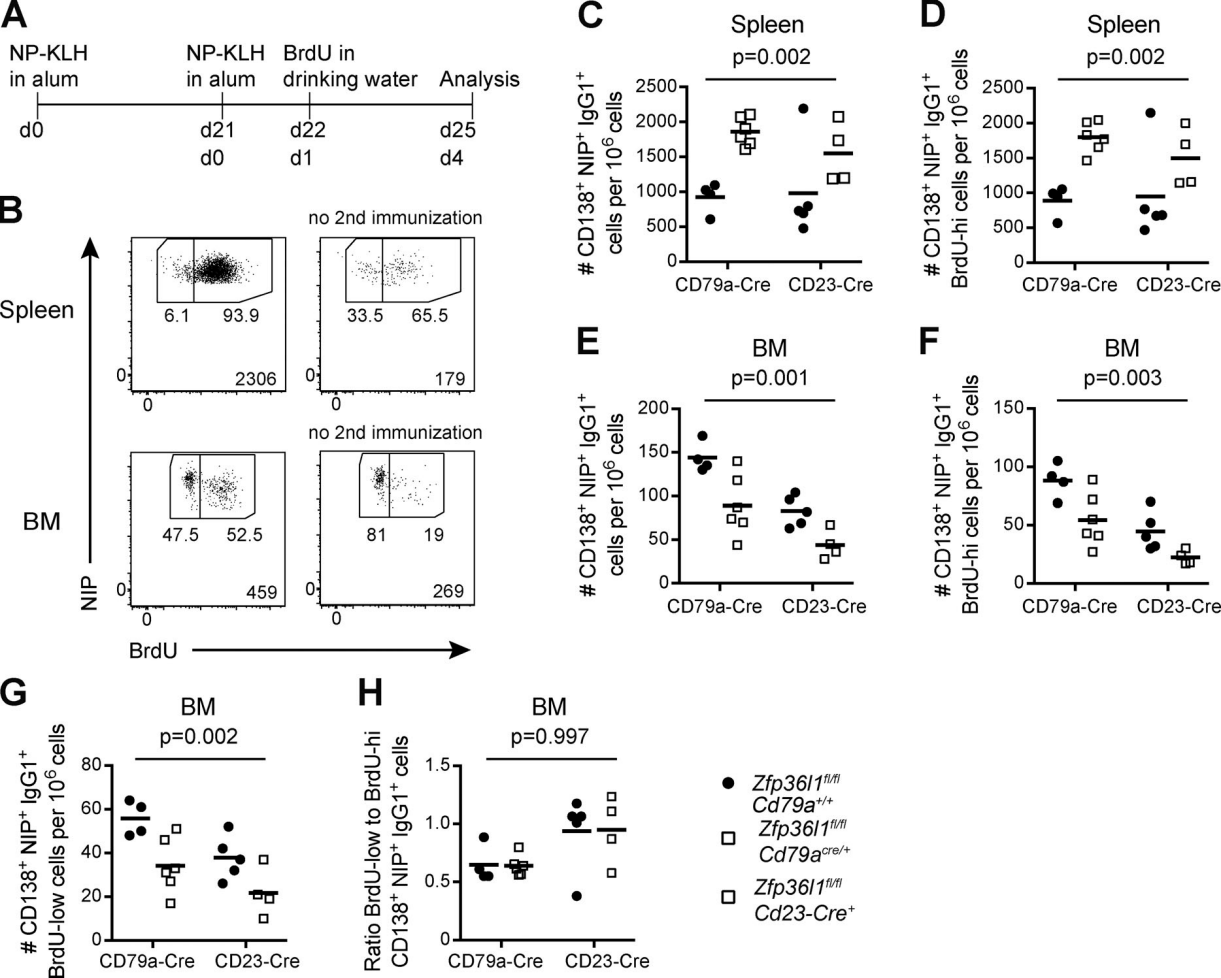
**Reduced number of Ag-specific ASCs in the BM of *Zfp36l1^fl/fl^ CD79a^Cre/+^* mice can be fully accounted for by defective migration of the newly generated ASCs to the BM.** **(A)** Schematic representation of the experimental setup for detection of the newly arrived NP-specific ASCs to the BM. **(B)** Representative flow cytometry plots gated as in Fig. 1 D on CD138^+^NIP^+^IgG1^+^ cells and used for detection of newly generated (BrdU-high) NP-specific ASCs and the established (BrdU-low) NP-specific ASCs in the spleen at day 4 following reimmunization with NP-KLH in alum (two top plots) and in the BM (two bottom plots). **(C and E)** Frequency of CD138^+^NIP^+^IgG1^+^ cells of *Zfp36l1^fl/fl^ CD79a^+/+^* (closed circles) and *Zfp36l1^fl/fl^ CD79a^Cre/+^* (open squares) or *Zfp36l1^fl/fl^ Cd23-Cre^+^* (open squares) mice at day 4 after reimmunization; the numbers of all CD138^+^NIP^+^IgG1^+^ cells in the spleen (C) and BM (E) are normalized to the numbers of all eFluor780^−^ cells in the sample. **(D, F, and G)** As above, but the populations of CD138^+^NIP^+^IgG1^+^ cells are split on the basis of BrdU staining into the BrdU-high NP-specific ASCs (D and F) and the BrdU-low NP-specific ASCs (G) and quantified in the spleen (D) and the BM (F and G). **(H)** The ratio of the number of BrdU-low NP-specific ASCs to the number of BrdU-high NP-specific ASCs in the BM. For all plots in all panels, each symbol represents data from an individual mouse, and lines represent means. For each Cre-expressing line, data are representative of two independent experiments (*n* ≥ 4; two-way ANOVA; the P values shown are for the main genotype effect). For all flow cytometry plots, the numbers next to the enclosed areas (gates) represent percentages of the cells falling into the gate, and the number in the bottom right corner shows the number of cells in the plot.

**Figure S2.**
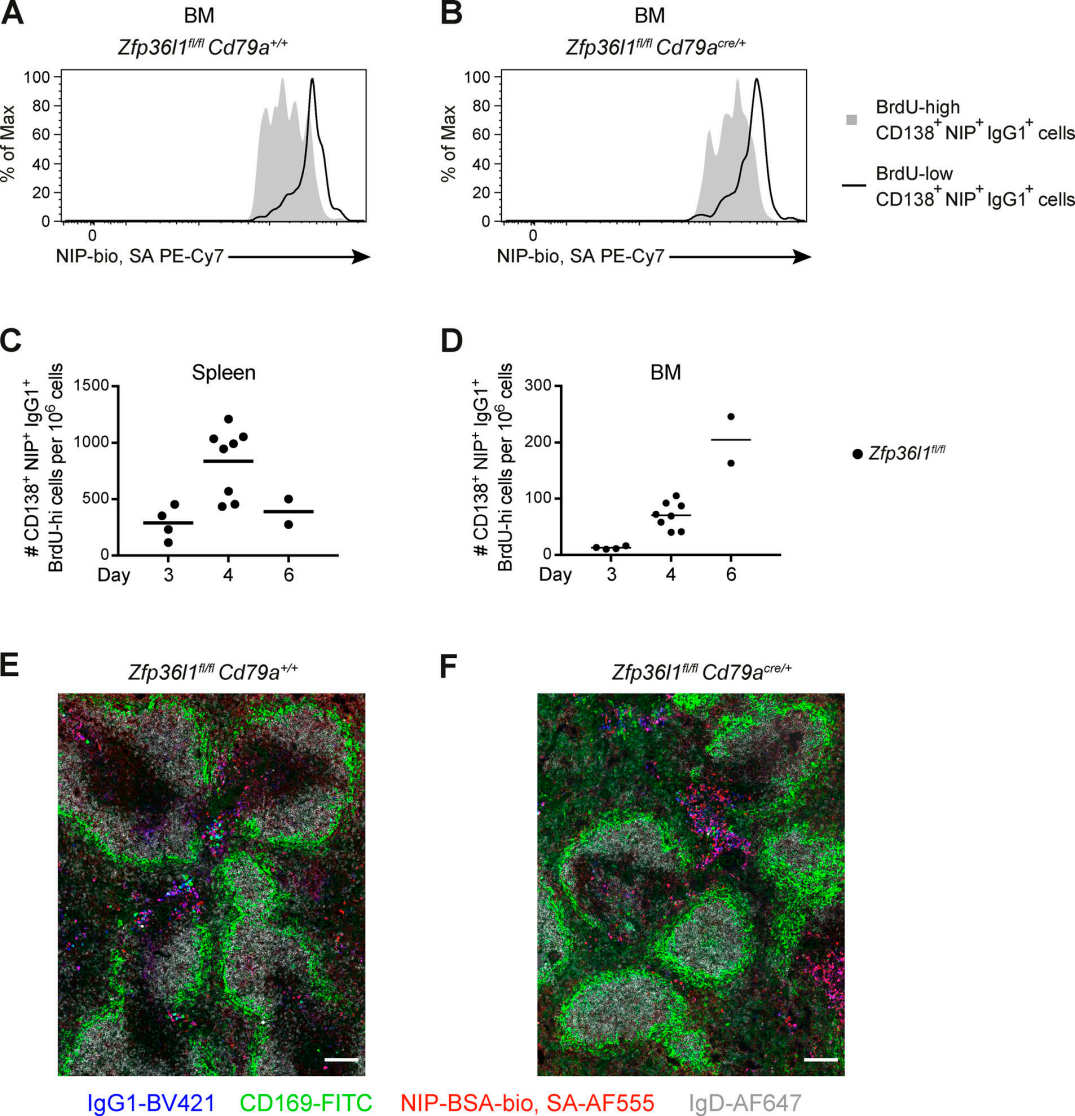
**Newly generated (BrdU-high) NP-specific ASCs do not appear in the BM before day 4 after reimmunization.** **(A and B)** An overlay of representative flow cytometry plots of BrdU-high (shaded histogram) and BrdU-low (black line) CD138^+^NIP^+^IgG1^+^ cells from the BM of *Zfp36l1^fl/fl^ CD79a^+/+^* (A) and *Zfp36l1^fl/fl^ CD79a^Cre/+^* (B) mice analyzed at day 4 after reimmunization. **(C and D)** Frequency of the newly generated (BrdU-high) CD138^+^NIP^+^IgG1^+^ cells in the spleen (C) and BM (D) *of Zfp36l1^fl/fl^ CD79a^+/+^* mice at days 3, 4, and 6 after reimmunization. The numbers of the BrdU-high CD138^+^NIP^+^IgG1^+^ cells are normalized to the numbers of all eFluor780^−^ cells in the sample. For all plots in all panels, each symbol represents data from an individual mouse, lines represent means, and data are representative of two independent experiments. **(E and F)** Immunofluorescence analysis of spleen sections from a *Zfp36l1^fl/fl^ CD79a^+/+^* mouse (E) and a *Zfp36l1^fl/fl^ CD79a^Cre/+^* mouse (F) at day 4 following secondary immunization. The sections were stained with anti-IgG1 (blue), anti-CD169 (MOMA-1, green), and anti-IgD (gray) antibody and NIP-BSA-bio Streptavidin Alexa Fluor 555 (red). The images are representative of sections taken from four mice for each of the genotypes. Original magnification, 20×; scale bars, 100 µm.

To discriminate between the outcomes of ASCs homing to the BM and of their subsequent survival, we monitored the appearance of NP-specific ASCs in the BM by giving mice BrdU in their drinking water the day after reimmunization and for the duration of the secondary response (Fig. 2 A). Incorporation of BrdU into the DNA of proliferating cells allows the identification of a population of antigen-specific ASCs elicited by the secondary immunization, which can be followed by flow cytometry. Consequently, staining of BM CD138^+^NIP^+^IgG1^+^ cells with anti-BrdU antibody revealed two distinct populations of ASCs: the BrdU-low population of quiescent ASCs established before day 22 and the BrdU-high ASCs that emerged after secondary immunization (Fig. 2 B). Interestingly, BrdU-low CD138^+^NIP^+^IgG1^+^ cells stained more strongly for NIP than BrdU-high cells, likely reflecting the increased production of antibody by ASCs established in the BM. This aspect of ASC maturation was not different between *Zfp36l1^fl/fl^ CD79a^Cre/+^* and *Zfp36l1^fl/fl^ CD79a^+/+^* mice (Fig. S2, A and B).

Tracing the BrdU-high CD138^+^NIP^+^IgG1^+^ cells in control mice over the course of the secondary response showed that in this system the newly generated ASCs do not appear in the BM until day 4 after reimmunization (Fig. S2, C and D). Analysis at this time point demonstrated an accumulation of the newly generated CD138^+^NIP^+^IgG1^+^ cells in the spleens of Zfp36l1 cKO mice (Fig. 2, C and D). The numbers of CD138^+^NIP^+^IgG1^+^ cells that migrated to the BM of Zfp36l1 cKO mice before and after day 24 (the last day before the appearance of elicited ASCs in the BM) were lower (Fig. 2, E–G), in agreement with previous observations (Fig. 1, B, C, and F). The difference in accumulation of ZFP36L1-sufficient and ZFP36L1-deficient ASCs in both the spleen and the BM was observed irrespective of whether the *CD79a^Cre^* or *Cd23-Cre* allele was used to delete the *Zfp36l1* gene (Fig. 2, C–G). Taken together, these data suggest that the reduction in ASC numbers in the BM results from the defective egress of ZFP36L1-deficient ASCs from the spleen.

4 d after secondary immunization, the population of the BM BrdU-low CD138^+^NIP^+^IgG1^+^ cells combined all NP-specific ASCs that were successful in migrating, differentiating, and surviving in the BM during the previous 24 d of the immune response, while the BrdU-high CD138^+^NIP^+^IgG1^+^ cells were the cells that migrated to the BM in the 24-h period before the analysis. Therefore, the ratio of BrdU-low to BrdU-high CD138^+^NIP^+^IgG1^+^ cells in the BM relates the number of established ASCs to the number of newly generated ASCs that arrived in the BM in a defined period of time immediately before the analysis. As this ratio is the same in Zfp36l1 cKO and control mice (Fig. 2 H), the observed reduction in the numbers of NP-specific ASCs in the BM can be fully explained by a failure of ZFP36L1-deficient ASCs to find their way to the BM.

### ZFP36L1-deficient ASCs have defective responses to S1P

**Figure 3.**
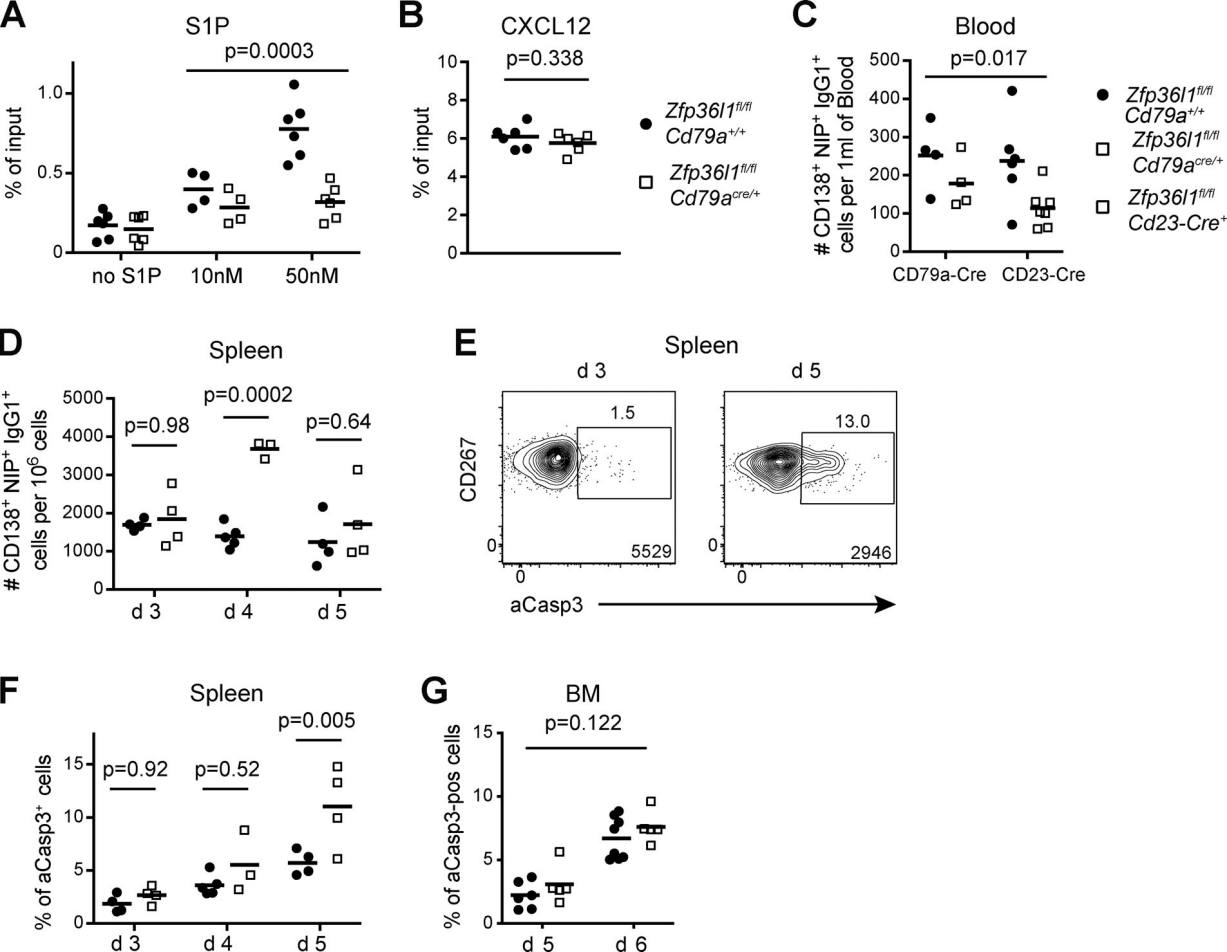
**Impaired accumulation of NP-specific ASCs in the BM of *Zfp36l1^fl/fl^ CD79a^Cre/+^* mice results from a delayed egress from the spleen.** **(A)** Quantification of a transwell migration assay of CD138^high^B220^low^ cells differentiated in vitro as described in Materials and methods. The number of cells migrated toward three different concentrations of S1P in the lower chamber was normalized to the number of identically gated cells in the input sample. The B cell cultures used in the transwell assay routinely contained 10–20% of CD138^high^B220^low^ cells (two-way ANOVA was performed on the data for 10 nM and 50 nM S1P; *n* = 4 and *n* = 6; data pooled from two or three independent experiments; each data point is a mean of technical duplicate measurements of independent biological samples, lines represent means, and the P value shown is for the main genotype effect for migration toward 10 nM and 50 nM S1P). **(B)** Quantification of a transwell migration assay performed as in A but toward CXCL12 (Student’s *t* test; *n* = 6; data pooled from three independent experiments; each data point is a mean of technical duplicate measurements of independent biological samples, and lines represent means). **(C)** Quantification of NP-specific ASCs (gated as eFluor780^−^TCRβ^−^CD138^+^Irf4^+^NIP^+^IgG1^+^ cells; full gating is depicted in Fig. S3 A) in blood of *Zfp36l1^fl/fl^ CD79a^+/+^* (closed circles) and *Zfp36l1^fl/fl^ CD79a^Cre/+^* (open squares) or *Zfp36l1^fl/fl^ Cd23-Cre^+^* (open squares) mice at day 3 after reimmunization with NP-KLH. For each Cre-expressing line, data are pooled from two independent experiments. For the plots in panels C, D, F, and G, each symbol represents data from an individual mouse, and lines represent means (*n* ≥ 4; two-way ANOVA; the P value shown is for the main factor “presence of Cre”). **(D)** Frequency of CD138^+^NIP^+^IgG1^+^ cells in the spleen of *Zfp36l1^fl/fl^ CD79a^+/+^* (closed circles) and *Zfp36l1^fl/fl^ CD79a^Cre/+^* (open squares) mice at days 3, 4, and 5 following reimmunization with NP-KLH expressed as numbers of CD138^+^NIP^+^IgG1^+^ cells normalized to the numbers of all eFluor780^−^ cells (*n* ≥ 3; two-way ANOVA with Sidak’s correction for multiple testing; the P values shown are for the genotype factor for separate days). **(E)** Representative flow cytometry plots gated as in Fig. 1 D on CD138^+^NIP^+^IgG1^+^ cells from *Zfp36l1^fl/fl^ CD79a^Cre/+^* mice 3 d (left plot) and 5 d (right plot) after reimmunization with NP-KLH and used to identify aCasp3^+^ cells. **(F and G)** Plots showing proportions of aCasp3^+^ cells in the population of CD138^+^NIP^+^IgG1^+^ cells identified as in Fig. 1 D, Fig. 3 E, and Fig. S3 B in the spleen (F) and BM (G) for *Zfp36l1^fl/fl^ CD79a^+/+^* (closed circles) and *Zfp36l1^fl/fl^ CD79a^Cre/+^* (open squares) mice taken at days 3, 4, 5, and 6 following reimmunization with NP-KLH. Data are representative of at least two independent experiments (*n* ≥ 3; two-way ANOVA with Sidak’s correction for multiple testing; the P values shown are for the genotype factor for separate days [F] or the P value shown is for the main genotype effect [G]). For all flow cytometry plots, the numbers next to the enclosed areas (gates) represent percentages of the cells falling into the gate, and the number in the bottom right corner shows the number of cells in the plot.

The migration of ASCs from secondary lymphoid organs to their long-term survival niches in the BM has been extensively studied. In the spleen, ASCs initially accumulate in a CXCR4-dependent manner near the sinusoids in the red pulp (Hargreaves et al., 2001). Their entry into the bloodstream has been shown to depend on S1PR1 (Kabashima et al., 2006). To assess the effects of ZFP36L1 deletion on the migratory capacity of ASCs, we used a transwell migration assay. Only 0.5–1% of the input ASCs are able to migrate toward S1P in these settings (Kabashima et al., 2006), making measurements of ex vivo antigen-specific ASC migration very challenging. Therefore, we used ASCs generated in vitro by activating follicular B (FoB) cells from the LNs of *Zfp36l1^fl/fl^ CD79a^+/+^* and *Zfp36l1^fl/fl^ CD79a^Cre/+^* mice with LPS for 96 h. Control ASCs showed a low but consistent migration that was enhanced by increasing the concentration of S1P. However, the ability of ZFP36L1-deficient ASCs to migrate to S1P was only slightly above the background level, and the cells were unresponsive to increased concentrations of S1P (Fig. 3 A). Notably, ZFP36L1-sufficient and ZFP36L1-deficient ASCs were equally efficient in migrating toward the CXCR4 ligand CXCL12 (Fig. 3 B). This observation demonstrated that the lack of response to S1P was specific rather than a reflection of a general failure to respond to a chemotactic cue.

Examination of splenic sections from *Zfp36l1^fl/fl^ CD79a^+/+^* and *Zfp36l1^fl/fl^ CD79a^Cre/+^* mice taken 4 d after reimmunization, when an accumulation of CD138^+^NIP^+^IgG1^+^ ASCs can be clearly detected by flow cytometry (Fig. 2 C), revealed cells with very high cytoplasmic staining for NIP and IgG1 that were consistently found in clusters in the red pulp in the mice of either genotype (Fig. S2, E and F), as would be expected for ASCs lacking the capacity to migrate to S1P (Kabashima et al., 2006). Consistent with intact CXCR4 responsiveness, we did not detect any abnormal accumulation of NIP^hi^IgG1^hi^ cells within the follicle or adjacent to the MZ, as was described for CXCR4-deficient ASCs (Hargreaves et al., 2001). A similarly unaltered localization of NIP^hi^IgG1^hi^ cells was observed in mice analyzed 14 d after primary immunization (data not shown). Overall, these results demonstrate that a buildup of CD138^+^NIP^+^IgG1^+^ ASCs in the spleens of *Zfp36l1^fl/fl^ CD79a^Cre/+^* mice can be attributed to defective signaling downstream of an S1P receptor.

### Defective egress of ASCs from the spleen of *Zfp36l1^fl/fl^ CD79a^Cre/+^* mice results in their increased cell death

**Figure S3.**
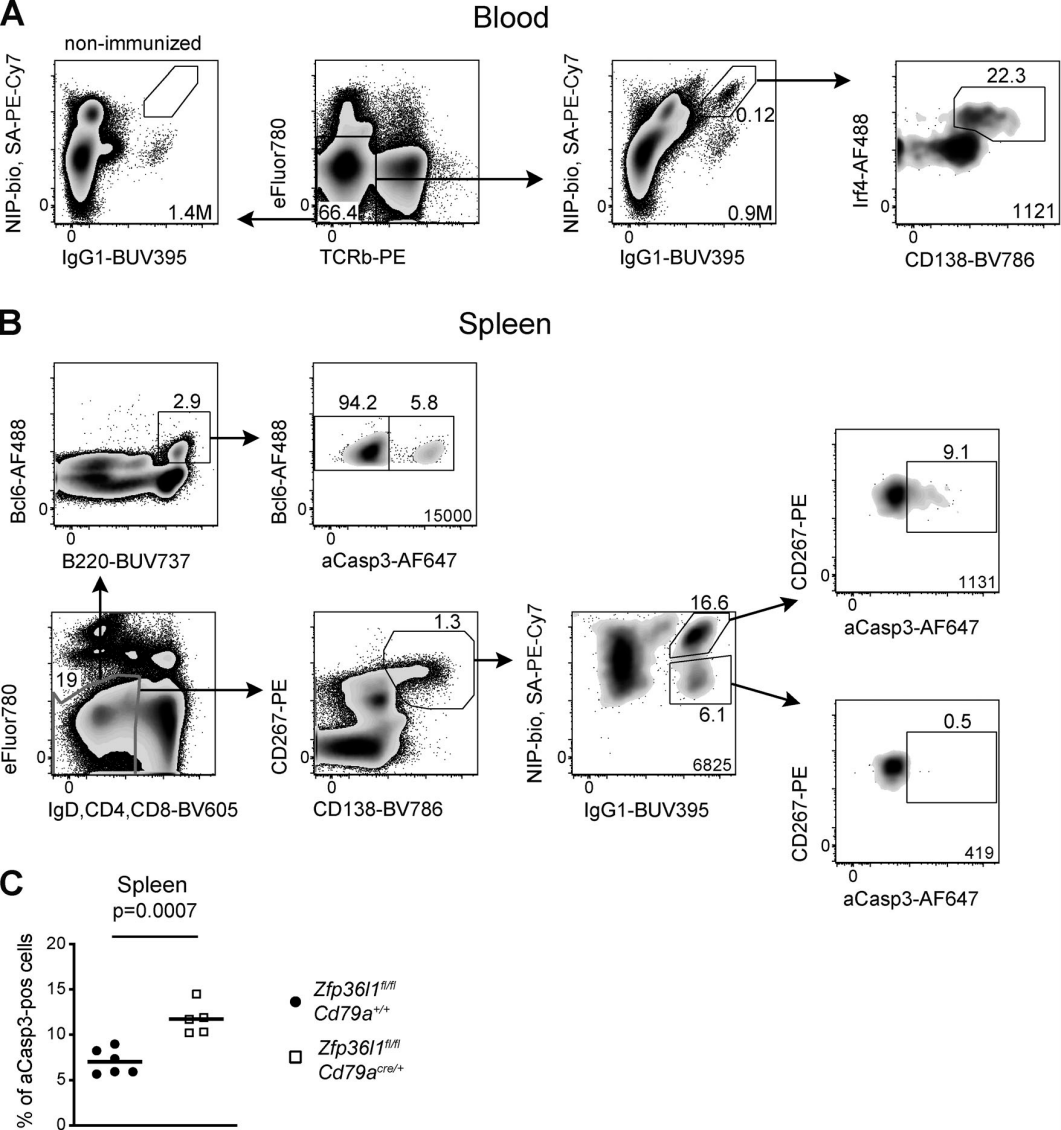
**The approach used in identification of NP-specific ASCs in the blood.** **(A)** A sequence of flow cytometry plots used to identify NP-specific ASCs in the blood. eFluor780^−^TCRb^−^ cells high for the intracellular NIP and IgG1 signals show a distinct population of CD138^+^Irf4^hi^ cells (rightmost plot). The number of such eFluor780^−^TCRb^−^CD138^+^Irf4^hi^NIP^+^IgG1^+^ cells per 1 ml blood was calculated as described in Materials and methods. **(B)** Flow cytometry plots showing proportions of aCasp3^+^ cells found in various splenic cell populations of the same mouse. eFluor780^−^CD4^−^CD8^−^IgD^−^CD138^+^CD267^+^ cells high for intracellular IgG1 but not for NIP were generally found to have very few aCasp3^+^ cells, presumably due to the fact that lower rates of their generation were not exceeding the rates of their elimination from the tissue. For all flow cytometry plots, the numbers next to the enclosed areas (gates) represent percentages of the cells falling into the gate, and the number in the bottom right corner shows the number of cells in the plot. Arrows denote the hierarchical relationship between the cell populations. **(C)** A plot showing proportions of aCasp3^+^ cells in the population of CD138^+^NIP^+^IgG1^+^ cells identified as in Fig. 1 D, Fig. 3 E, and Fig. S3 B in the spleens of *Zfp36l1^fl/fl^ CD79a^+/+^* (closed circles) and *Zfp36l1^fl/fl^ CD79a^Cre/+^* (open squares) mice taken at day 5 following reimmunization with NP-KLH in an independent experiment. Each symbol represents data from an individual mouse, and lines represent means (*n* ≥ 5; the P value is for a Student’s *t* test). Pos, positive.

Impaired egress from the spleen should result in a reduced number of ASCs entering the bloodstream. To test this prediction directly, we determined the number of CD138^+^NIP^+^IgG1^+^ cells in the blood of control and Zfp36l1 cKO mice 3 d after reimmunization by flow cytometry. Since the surface abundance of CXCR4 and CD267 is low on blood CD138^+^NIP^+^IgG1^+^ cells, we used the transcription factor IRF4 as an additional marker for ASCs (Klein et al., 2006; Sciammas et al., 2006; Fig. S3 A). We found a twofold reduction in the number of CD138^+^NIP^+^IgG1^+^ cells in the blood of Zfp36l1 cKO mice independent of the Cre system used to drive deletion of the *Zfp36l1^fl^* allele (Fig. 3 C). Importantly, the number of CD138^+^NIP^+^IgG1^+^ cells recovered on day 3 from the spleens of *Zfp36l1^fl/fl^ CD79a^Cre/+^* mice was similar to that in control animals (Fig. 3 D), arguing against an accelerated generation of ZFP36L1-deficient ASCs as a cause for their marked accumulation on day 4 (Fig. 3 D). By day 5, however, the numbers of NP-specific ASCs in Zfp36l1 cKO mice was not different from that of controls (Fig. 3 D).

Because the number of antigen-specific ASCs in the BM of Zfp36l1 cKO mice at days 5 and 6 following reimmunization (Fig. 1 F) was lower than that of controls, it is likely that the delayed egress from the spleen resulted in their death, either in the spleen or en route to the BM. To understand if CD138^+^NIP^+^IgG1^+^ cells in *Zfp36l1^fl/fl^ CD79a^Cre/+^* mice undergo apoptosis at increased rates, we enumerated dying ASCs identified as being positive for active caspase 3 (aCasp3^+^). Normally, the dead cells are quickly eliminated from the spleen so that only ∼5% of aCasp3^+^ cells can be found even in cell populations with a high turnover, such as GC B cells (Mayer et al., 2017; Fig. S3 B). From days 3 to 5 following reimmunization, control mice showed a progressive buildup in the proportion of aCasp3^+^ cells among CD138^+^NIP^+^IgG1^+^ ASCs (Fig. 3, E and F), demonstrating that transition of ASCs to the bloodstream is naturally inefficient. *Zfp36l1^fl/fl^ CD79a^Cre/+^* mice displayed an equal accumulation of aCasp3^+^ cells in the splenic CD138^+^NIP^+^IgG1^+^ compartment at day 3. However, at day 5, the day after the peak response in Zfp36l1 cKO mice (Fig. 3 D), ∼10% of all NP-specific ASCs were aCasp3^+^, a twofold increase compared with control animals (Fig. 3 F and Fig. S3 C). Notably, the percentage of apoptotic cells in the BM population of CD138^+^NIP^+^IgG1^+^ cells was similar between *Zfp36l1^fl/fl^ CD79a^+/+^* and *Zfp36l1^fl/fl^ CD79a^Cre/+^* mice (Fig. 3 G). Cell death among BM NP-specific ASCs showed an acceleration from days 5 to 6, demonstrating that in the BM as well as the spleen, the appearance of aCasp3^+^ cells can overcome the processes leading to their elimination. Nevertheless, ZFP36L1-deficient CD138^+^NIP^+^IgG1^+^ ASCs do not exhibit a noticeably higher predisposition to cell death once they reach the BM. Overall, these data demonstrate that a generally inefficient egress of ASCs from the spleen is associated with a high incidence of cell death and that ZFP36L1 promotes their prompt transition into the bloodstream, thereby reducing inherently high attrition rates.

### ZFP36L1 regulates genes involved in S1P receptor signaling

To establish candidate transcripts regulated directly by ZFP36l1, we identified RNA molecules bound to ZFP36L1 at nucleotide resolution using individual-nucleotide resolution cross-linking and immunoprecipitation (iCLIP). Because the iCLIP protocol for ZFP36L1 requires immunoprecipitation of a low-abundance protein and application of highly stringent washes to identify specific interactions, it necessitates the use of a large number of cells and cannot be applied to ASCs. Therefore, we used a previously published iCLIP dataset obtained for LN FoB cells activated with LPS for 48 h (Galloway et al., 2016) and RNA sequencing (RNA-seq) on similarly activated FoB cells from *Zfp36l1^fl/fl^ CD79a^Cre/+^* and *Zfp36l1^fl/fl^ CD79a^+/+^* mice.

**Figure 4.**
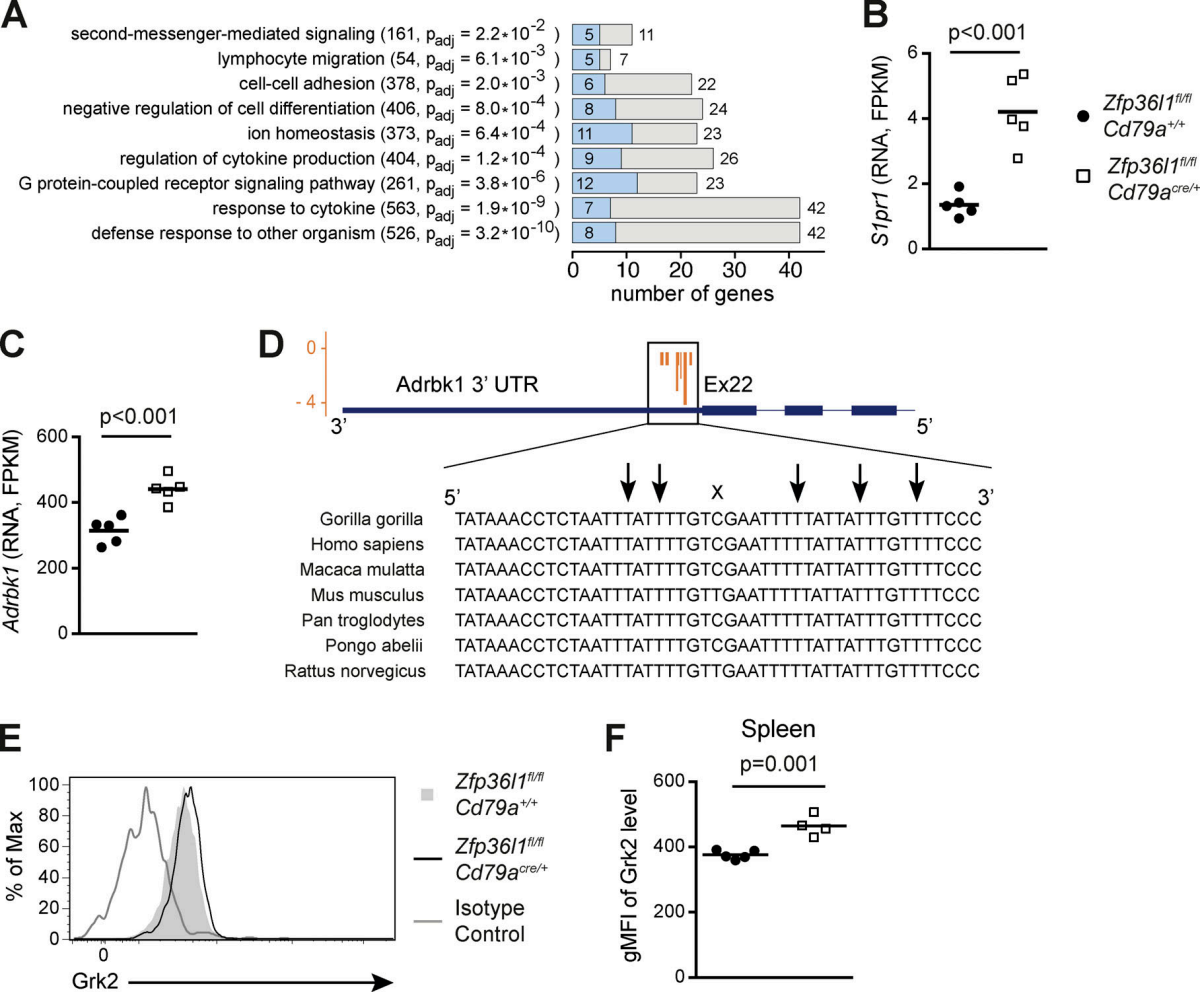
**ZFP36L1-deficient ASCs contain higher levels of GRK2.** **(A)** GO terms enriched in gene set enrichment analysis performed on the genes differentially expressed (DE) in ZFP36L1-deficient and ZFP36L1-sufficient B cells activated with LPS. For each term, the numbers in parentheses are the numbers of all genes in the term followed by the adjusted P (p_adj_) value. The numbers outside the gray bars represent the number of DE genes (p_adj_ < 0.05; fold change >1.5), and the numbers inside the blue bars represent the numbers of direct targets of ZFP36L1 identified by iCLIP (p_adj_ < 0.05), all overlapping with the corresponding GO terms. Under the conditions used, activated B cells undergo at most three cell divisions and do not contain any CD138^high^B220^low^ cells. **(B and C)** Abundance of mRNA for *S1pr1* gene for S1PR1 (B) and *Adrbk1* gene encoding GRK2 kinase (C) expressed as FPKM in the LPS-activated B cells from *Zfp36l1^fl/fl^ CD79a^+/+^* (closed circles) and *Zfp36l1^fl/fl^ CD79a^Cre/+^* (open squares) mice. Each symbol represents data from B cells of an individual mouse, lines represent means, and P values are adjusted by the Benjamini-Hochburg method calculated with DESeq2. **(D)** Conservation of the sequence surrounding cross-linked nucleotides in the 3′ UTR of *Adrbk1* transcripts among primates and rodents. Cross-linked nucleotides were visualized using the UCSC Genome Browser (http://genome.ucsc.edu). In the gene diagram, the blue boxes denote exons, the thinner line denotes 3′ UTR, and the thin lines show introns. Arrows identify nucleotides cross-linked to ZFP36L1, and X indicates a nonconserved nucleotide. **(E)** An overlay of representative flow cytometry plots gated on CD138^+^NIP^+^IgG1^+^ cells from the spleens of *Zfp36l1^fl/fl^ CD79a^+/+^* and *Zfp36l1^fl/fl^ CD79a^Cre/+^* mice analyzed 3 d after reimmunization. Rabbit polyclonal antibody against GRK2 was conjugated to Alexa Fluor 647. **(F)** A plot showing geometric mean fluorescent intensities (gMFIs) for the GRK2 signal in the population of splenic CD138^+^NIP^+^IgG1^+^ cells as in panel E for *Zfp36l1^fl/fl^ CD79a^+/+^* (closed circles) and *Zfp36l1^fl/fl^ CD79a^Cre/+^* (open squares) mice. Each symbol represents data from an individual mouse, and lines represent means. Data for independent experiments are shown in Fig. S4 B (*n* ≥ 4 per experiment; the P value is for a Student’s *t* test).

Analysis of differences in transcript abundance by DESeq2 followed by pathway-enrichment analysis of 272 differentially expressed genes (change >1.5-fold; adjusted P value <0.05; Table S1) showed that many genes with altered transcript abundance are involved in the regulation of cell motility, adhesion, and migration (Fig. 4 A). Depending on the gene ontology (GO) term, the binding of ZFP36L1 to the cognate transcripts can be detected by iCLIP for up to half of the genes in the term (Fig. 4 A, blue bars). Notably, we found that the transcripts for both S1PR1 and for the kinase G protein–coupled receptor kinase 2 (GRK2) encoded by the gene *Adrbk1* were more abundant in B cells from Zfp36l1 cKO mice (Fig. 4, B and C). While iCLIP analysis failed to uncover interaction between ZFP36L1 and *S1pr1* transcripts, we note several AU-rich elements in the 3′ UTR of *S1pr1* mRNA, indicating a potential for direct regulation by ZFP36L1. A very low abundance of *S1pr1* mRNA in activated B cells likely renders ZFP36L1 binding undetectable and makes the exact nature of *S1pr1* regulation in primary B cells difficult to ascertain. The ZFP36L1-binding sequence in the 3′ UTR of *Adrbk1* mRNA identified by iCLIP analysis exhibits a very high degree of conservation between mammalian species (Fig. 4 D), further substantiating direct regulation of the expression of this kinase by ZFP36L1.

**Figure S4.**
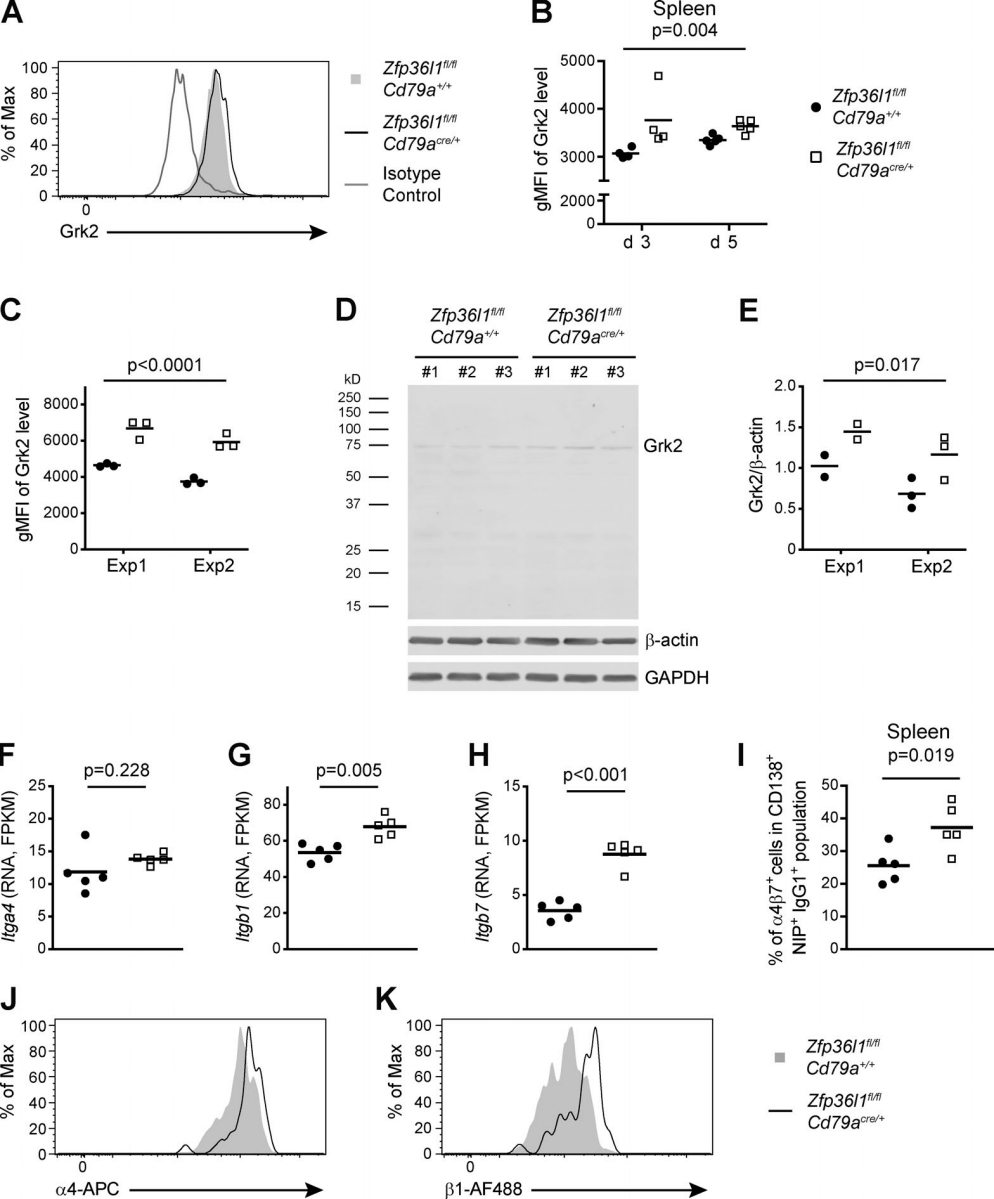
**GRK2 abundance is increased in NP-specific splenic ASCs in *Zfp36l1^fl/fl^CD79a^Cre/+^* mice.** **(A)** An overlay of representative flow cytometry plots gated on CD138^+^NIP^+^IgG1^+^ cells from the spleens of *Zfp36l1^fl/fl^ CD79a^+/+^* and *Zfp36l1^fl/fl^ CD79a^Cre/+^* mice analyzed at days 3 and 5 after reimmunization. Antibody against GRK2 used in these experiments was from Cell Signaling Technology and was used in combination with the secondary Alexa Fluor 647–conjugated donkey anti-rabbit IgG antibody. **(B)** A plot showing geometric mean fluorescent intensities (gMFIs) for GRK2 signal in the population of CD138^+^NIP^+^IgG1^+^ cells. Each symbol represents data from an individual mouse, and lines represent means. Data are from independent experiments performed in confirmation of Fig. 4 F (*n* ≥ 4 per experiment; two-way ANOVA; the P value shown is for the main genotype effect). **(C)** A plot showing gMFIs for GRK2 signal in the population of CD138^high^B220^low^ cells differentiated in vitro. GRK2 was detected with antibody from OriGene followed by the secondary Alexa Fluor 647–conjugated donkey anti-rabbit IgG antibody. Each symbol represents data from an individual mouse, and lines represent means. Data are from two independent experiments (*n* = 3 per experiment; two-way ANOVA; the P value shown is for the main genotype effect). **(D)** Representative Western blot of total protein extracts of CD138^high^B220^low^ cells differentiated in vitro and sorted by a flow cytometer as described in Materials and methods. The upper panel shows the membrane incubated with antibody against GRK2, and the lower panels show the same membrane incubated with antibody against β-actin and against GAPDH. **(E)** A plot showing relative abundance of GRK2 as determined by a ratio of the signal for the band corresponding to GRK2 to the signal for β-actin (a comparable result was obtained with GRK2 signal normalized to that of GAPDH; data not shown). Each symbol represents data from an individual mouse, and lines represent means (*n* ≥ 2 per experiment; two-way ANOVA; the P value shown is for the main genotype effect). **(F–H)** Abundance of mRNA for the *Itga4* (F), *Itgb1* (G), and *Itgb7* (H) genes in LPS-activated B cells from *Zfp36l1^fl/fl^ CD79a^+/+^* (closed circles) and *Zfp36l1^fl/fl^ CD79a^Cre/+^* (open squares) mice. Each symbol represents data from B cells of an individual mouse, and lines represent means. P values calculated with DESeq2 are adjusted by the Benjamini-Hochburg method. **(I)** Percentages of cells with a high integrin α4β7 surface abundance in the splenic populations of CD138^+^NIP^+^IgG1^+^ cells in *Zfp36l1^fl/fl^ CD79a^+/+^* (closed circles) and *Zfp36l1^fl/fl^ CD79a^Cre/+^* (open squares) mice at day 14 following primary immunization. Each symbol represents data from an individual mouse, and lines represent means. Data are representative of at least two independent experiments (*n* = 5; the P value is for a Student’s *t* test). **(J and K)** An overlay of representative flow cytometry plots showing surface expression of the integrin chains α4 (J) and β1 (K) on CD138^+^NIP^+^IgG1^+^ cells from the spleens of *Zfp36l1^fl/fl^ CD79a^+/+^* (shaded histogram) and *Zfp36l1^fl/fl^ CD79a^Cre/+^* (black line) mice analyzed 42 d after immunization.

To investigate if ZFP36L1-deficient splenic ASCs have increased expression of GRK2, the splenocytes taken 3 d after reimmunization were stained intracellularly with anti-GRK2 antibodies. CD138^+^NIP^+^IgG1^+^ ASCs from Zfp36l1 cKO mice demonstrated a small but consistent increase in the abundance of GRK2 protein (Fig. 4, E and F), which was validated in independent experiments with another antibody raised against a different epitope of GRK2 (Fig. S4, A and B). A similar increase in GRK2 level in the absence of ZFP36L1 was demonstrated for in vitro–generated ASCs by both flow cytometry (Fig. S4 C) and Western blotting (Fig. S4, D and E). GRK2 is primarily thought of as a negative regulator of S1P receptor signaling in lymphocytes (Arnon et al., 2011), and its elevated abundance can account for the defective chemotactic response of ZFP36L1-deficient ASCs to S1P. Dysregulation of other genes in ZFP36L1 cKO mice cannot be excluded as a factor in impaired egress of ASCs from the spleen, but their contribution is likely to be obscured by the impact of increased GRK2.

### Regulation of integrin expression on ASCs by ZFP36L1 at early and late stages of GC response

**Figure 5.**
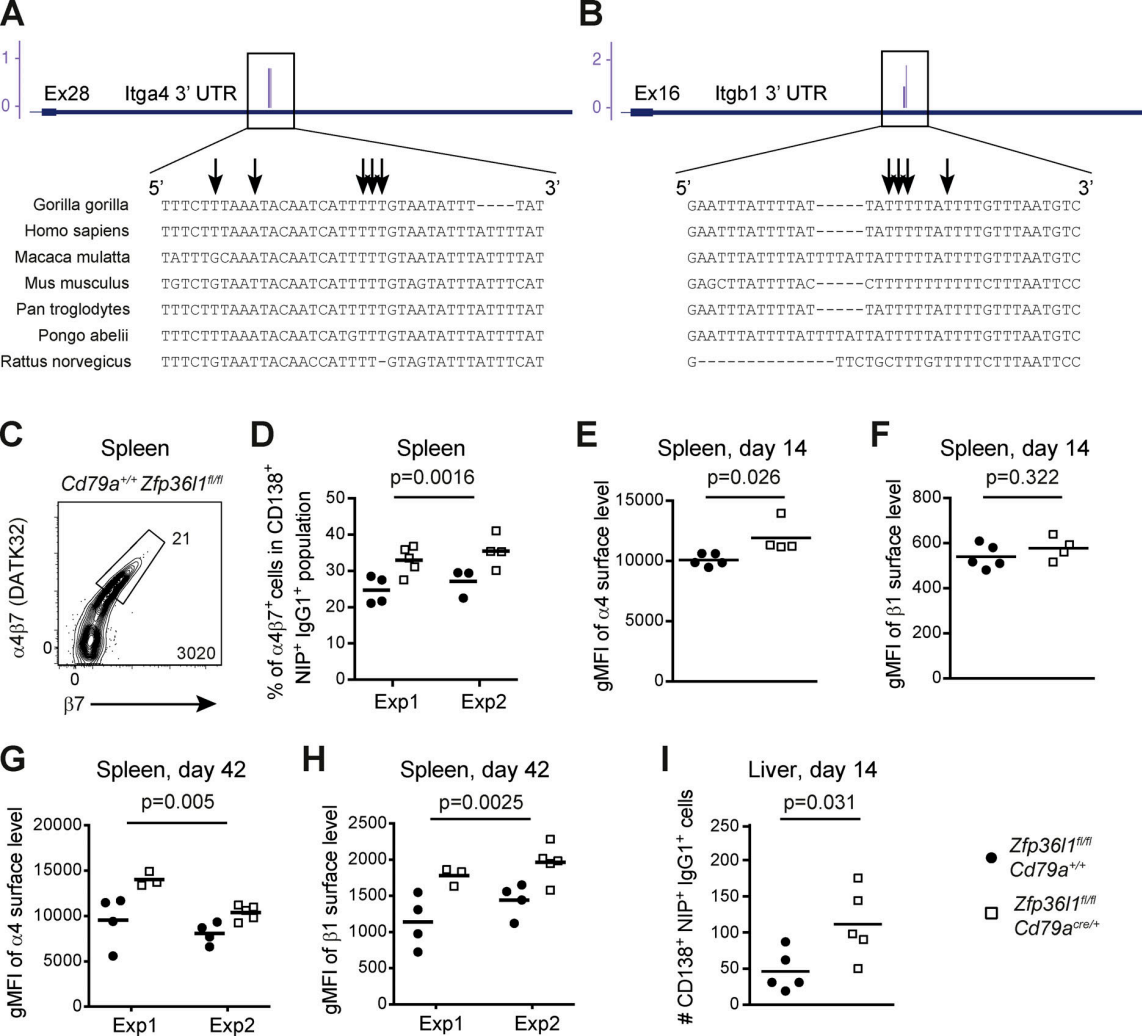
**Increase in the surface expression of the integrin chains α4 and β1 on splenic ASCs in *Zfp36l1^fl/fl^ CD79a^Cre/+^* mice becomes more pronounced on ASCs generated late in the immune response and is associated with a partial retention of ASCs in the liver.** **(A and B)** Conservation of the sequence surrounding cross-linked nucleotides in the 3′ UTRs of the *Itga4* (A) and *Itgb1* (B) transcripts among a selection of primates and rodents. Cross-linked nucleotides were visualized using the UCSC Genome Browser (http://genome.ucsc.edu). In the gene diagram, the blue boxes denote exons, the thinner lines denote 3′ UTRs, and the thin lines show introns. Arrows identify nucleotides cross-linked to ZFP36L1. **(C)** Representative flow cytometry plot gated as in Fig. 1 D on CD138^+^NIP^+^IgG1^+^ cells from the spleen of *Zfp36l1^fl/fl^ CD79a^+/+^* mice at day 4 after reimmunization. The number close to the enclosed area shows the percentage of α4β7-high NP-specific ASCs and the number in the bottom right corner shows the number of cells in the plot. **(D)** Percentages of cells with a high α4β7 surface abundance in the splenic populations of CD138^+^NIP^+^IgG1^+^ cells in *Zfp36l1^fl/fl^ CD79a^+/+^* (closed circles) and *Zfp36l1^fl/fl^ CD79a^Cre/+^* (open squares) mice at day 4 following reimmunization (*n* ≥ 3; two-way ANOVA; the P value shown is for the main genotype effect). **(E–H)** Plots showing geometric mean fluorescent intensities (gMFIs) for the surface integrin α4 (E and G) and β1 (F and H) signals on the population of CD138^+^NIP^+^IgG1^+^ cells from the spleens of *Zfp36l1^fl/fl^ CD79a^+/+^* (closed circles) and *Zfp36l1^fl/fl^ CD79a^Cre/+^* (open squares) mice at day 14 (E and F) and day 42 (G and H) after primary immunization (*n* ≥ 3 per experiment; the P values are for a Student’s *t* test [E and F] or for the main genotype effect in a two-way ANOVA on logarithmically transformed data [G and H]). Where it is not indicated otherwise data are representative of at least two independent experiments. Exp, experiment. **(I)** Enumeration of CD138^+^NIP^+^IgG1^+^ cells (gated as eFluor780^−^TCRβ^−^CD138^+^CD267^+^NIP^+^IgG1^+^ cells) in the livers of *Zfp36l1^fl/fl^ CD79a^+/+^* (closed circles) and *Zfp36l1^fl/fl^ CD79a^Cre/+^* (open squares) mice at day 14 after primary immunization; the numbers of CD138^+^NIP^+^IgG1^+^ cells are calculated for whole livers. For all plots in all panels, each symbol represents data from an individual mouse, and lines represent means (the P value is for a Student’s *t* test; *n* = 5; data pooled from two independent experiments).

Integrins have been extensively documented to play an important role in the homing of cells to the BM. The integrin dimer α4β1 and its ligand VCAM-1 are thought to be dominant molecules as demonstrated for hematopoietic stem cells and B cells (Berlin-Rufenach et al., 1999; Koni et al., 2001; Leuker et al., 2001), while a requirement for the α4β7 dimer has also been reported (Katayama et al., 2004). We found that mRNA for the integrin chains α4, β1, and β7 was more abundant in ZFP36L1-deficient B cells (Fig. S4, F–H). The transcripts for the α4 and β1 chains were also identified by iCLIP analysis as bound by ZFP36L1 at highly conserved regions of their 3′ UTRs (Fig. 5, A and B); however, binding to the β7 mRNA was undetectable, suggesting an indirect regulation of the β7 chain by ZFP36L1.

We consistently detected an increased number of CD138^+^NIP^+^IgG1^+^ ASCs with a high surface level of the α4β7 dimer in the spleens of Zfp36l1 cKO mice (Fig. 5, C and D; and Fig. S4 I). As expected, the α4 integrin chain was elevated on ZFP36L1-deficient ASCs 14 d after immunization (Fig. 5 E), but we observed no difference in expression of the β1 chain (Fig. 5 F). The latter finding was somewhat perplexing, and we hypothesized that regulation of the integrin chain β1 by ZFP36L1 becomes apparent later in the immune response. Indeed, analysis of the β1 surface level on splenic NP-specific ASCs in *Zfp36l1^fl/fl^ CD79a^Cre/+^* mice at day 42 demonstrated a modulation of its expression by ZFP36L1 (Fig. 5 H and Fig. S4 K), as was the case for the integrin α4 chain (Fig. 5 G and Fig. S4 J). Thus, ZFP36L1 limits surface levels of the integrin chains α4, β1, and β7 on splenic ASCs, with regulation of the β1 chain becoming obvious only later in the response.

VCAM-1 and fibronectin, ligands for the α4β1 and α4β7 dimers, can be detected in the red pulp (Lu and Cyster, 2002; Liakka and Autio-Harmainen, 1992), potentially contributing to retention of splenic ASCs with aberrantly high surface expression of these integrins. Furthermore, because blood from the spleen flows into the hepatic portal vein and through the liver, such CD138^+^NIP^+^IgG1^+^ cells may also adhere more strongly to the endothelial cells lining the blood vessels and enter the liver. To explore this possibility, we enumerated NP-specific ASCs in the livers of mice 14 d after immunization. The livers of *Zfp36l1^fl/fl^ CD79a^Cre/+^* mice showed a twofold increase in the number of CD138^+^NIP^+^IgG1^+^ cells compared with the livers of *Zfp36l1^fl/fl^ CD79a^+/+^* mice (Fig. 5 I), demonstrating that a higher expression of α4β1 and α4β7 dimers on the surface of splenic ASCs in Zfp36l1 cKO mice may further reduce their chances of successfully reaching the BM.

### Dynamic regulation of CXCR4 and integrin expression on ASCs during migration to the BM

**Figure S5.**
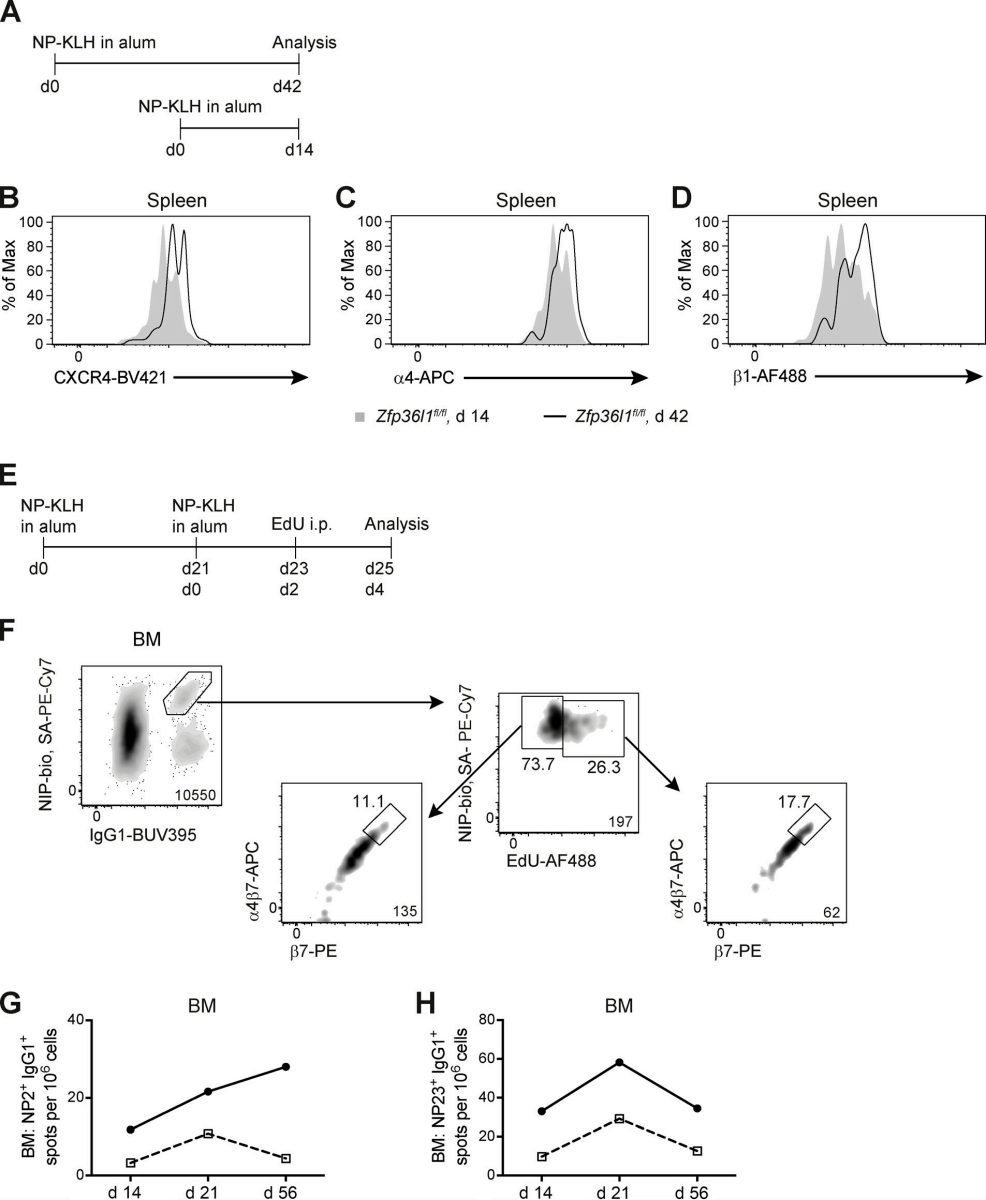
**Dynamic regulation of the integrin chains α4, β1, and β7 and of CXCR4 on ASCs.** **(A)** Schematic representation of the experimental setup for comparison of NP-specific ASCs generated at different times during the immune response. **(B–D)** An overlay of representative flow cytometry plots showing surface expression of CXCR4 (B) and of the integrin chains α4 (C) and β1 (D) on CD138^+^NIP^+^IgG1^+^ cells from the spleens of *Zfp36l1^fl/fl^* mice analyzed 14 d (shaded histogram) and 42 d (black line) after immunization. **(E)** Schematic representation of the experimental setup for pulse labeling of ASCs with EdU and detection of the newly arrived NP-specific ASCs to the BM. **(F)** Representative flow cytometry plots gated as in Fig. 1 D on CD138^+^NIP^+^IgG1^+^ cells and used for identification of the EdU-low and EdU-high NP-specific ASCs in the BM at day 4 following reimmunization. For all flow cytometry plots, the numbers next to the enclosed areas (gates) represent percentages of the cells falling into the gate, and the number in the bottom right corner shows the number of cells in the plot. Arrows denote the hierarchical relationship between the cell populations. **(G and H)** ELISPOT analysis of the BM in *Zfp36l1^fl/fl^ CD79a^+/+^* (closed circles) and *Zfp36l1^fl/fl^ CD79a^Cre/+^* (open squares) mice for ASCs secreting anti-NP_2_ (high affinity) IgG1 antibody (G) and for ASCs secreting anti-NP_23_ (high and low affinity) IgG1 antibody (H). The data in Fig. 1, B and C are presented as lines connecting the means of groups for each time point for each genotype.

**Figure 6.**
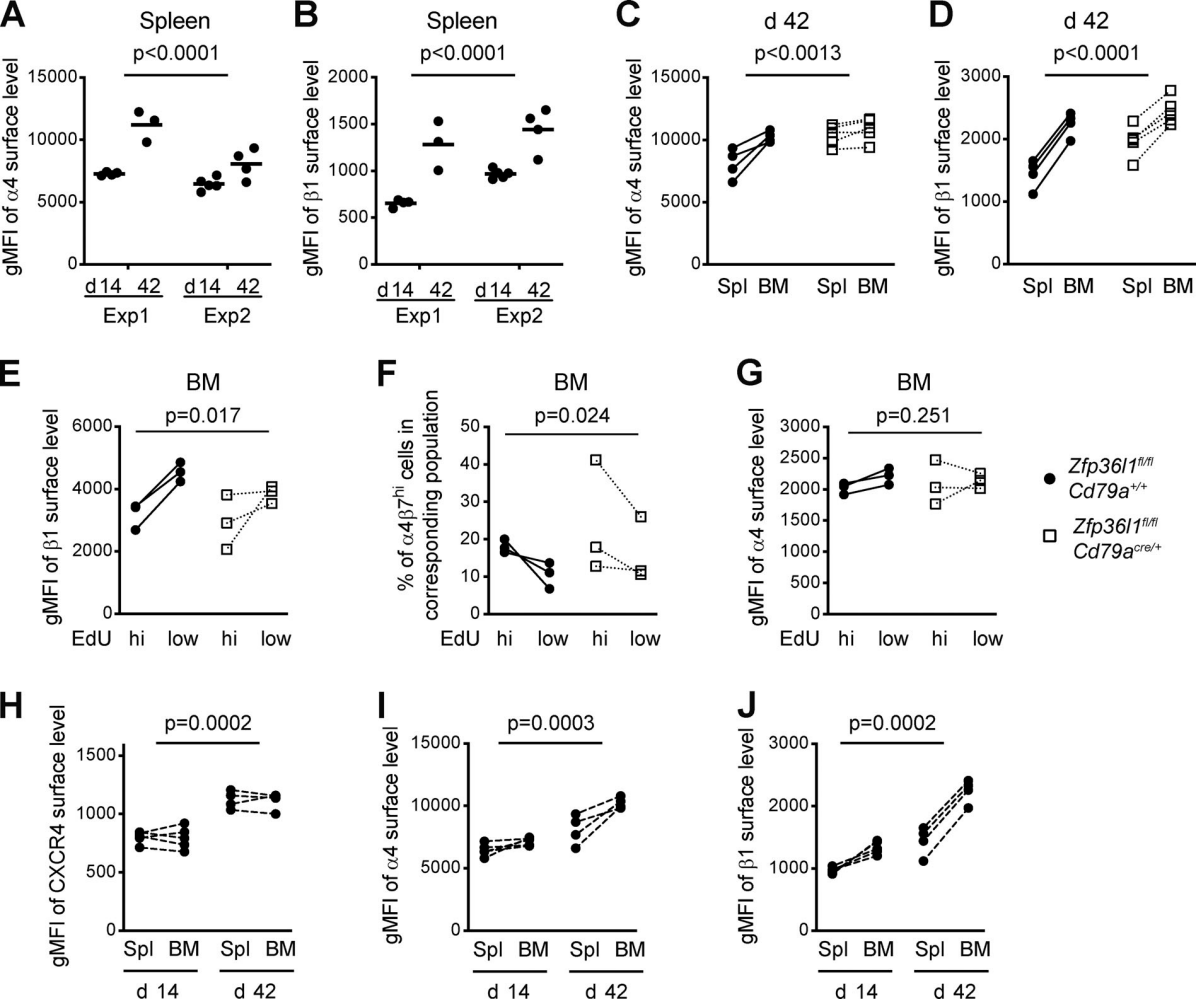
**BM CD138^+^NIP^+^IgG1^+^ cells display elevated levels of the integrins α4 and β1 on their surface compared with splenic CD138^+^NIP^+^IgG1^+^ cells from the same animal.** **(A and B)** Plots showing geometric mean fluorescent intensities (gMFIs) for surface integrin α4 (A) and β1 (B) signals on the population of CD138^+^NIP^+^IgG1^+^ cells from the spleens of *Zfp36l1^fl/fl^ CD79a^+/+^* mice at days 14 and 42. The staining and analysis were performed on the same day with the same flow cytometer settings; the data for two independent experiments are shown (*n* ≥ 3 per experiment; two-way ANOVA on logarithmically transformed data; the P values shown are for the day factor). **(C and D)** Plots showing gMFI for surface integrin α4 (C) and β1 (D) signals on the population of CD138^+^NIP^+^IgG1^+^ cells from the spleens and BM of individual *Zfp36l1^fl/fl^ CD79a^+/+^* (closed circles) and *Zfp36l1^fl/fl^ CD79a^Cre/+^* (open squares) mice analyzed at day 42 (*n* ≥ 4; two-way ANOVA; the P values shown are for the organ factor). **(E–G)** Plots showing percentages of cells with a high α4β7 surface abundance (F) and gMFI for surface level of the integrin chains β1 (E) and α4 (G) among both EdU-high NP-specific ASCs and EdU-low NP-specific ASCs in the BM of mice of both genotypes at day 4 after reimmunization. Populations were identified as depicted in Fig. S5 F (*n* = 3; two-way ANOVA; the P value shown is for the factor “type of ASC”). **(H–J)** Plots showing gMFIs for CXCR4 (H) and for the surface integrin α4 (I) and β1 (J) signals on the population of CD138^+^NIP^+^IgG1^+^ cells from the spleen and BM of individual *Zfp36l1^fl/fl^ CD79a^+/+^* mice at day 14 and day 42 after primary immunization (*n* ≥ 4 per time point; two-way ANOVA; the P values shown are for the day factor). For all plots in all panels, each symbol represents data from an individual mouse, and lines represent means. Where it is not indicated otherwise, data are representative of at least two independent experiments. Exp, experiment; Spl, spleen; hi, high.

As the degree of integrin β1 control by ZFP36L1 depends on the time point in the primary immune response when ASCs are generated (Fig. 5, F and H), we hypothesized that expression of this integrin can increase progressively with maturation of the GCs. To test this hypothesis, we simultaneously analyzed mice that had an immune response lasting for 14 and 42 d (Fig. S5 A). Indeed, the surface abundance of the integrins α4 and β1 was higher on splenic CD138^+^NIP^+^IgG1^+^ ASCs recovered 42 d after immunization than on ASCs recovered at day 14 (Fig. 6, A and B; and Fig. S5, C and D).

BM CD138^+^NIP^+^IgG1^+^ ASCs had greater surface expression of the integrins α4 and β1 than CD138^+^NIP^+^IgG1^+^ cells from the spleen of the same mouse independently of the genotype (Fig. 6, C and D). Regulation of integrin expression upon ASC transition to the BM was probed directly by pulse labeling developing ASCs with 5-ethynyl-2′-deoxyuridine (EdU) 2 d after reimmunization and analysis of BM cells 2 d later (Fig. S5 E). A population of newly immigrated EdU-high CD138^+^NIP^+^IgG1^+^ ASCs (Fig. S5 F) had a lower level of integrin β1 on the surface compared with that of the established EdU-low ASCs (Fig. 6 E). Interestingly, the α4β7 dimer displayed an opposite pattern of surface expression (Fig. 6 F), while the levels of integrin α4 appeared to be largely unchanged (Fig. 6 G). Together, these results demonstrate that maturation of ASCs in the BM is associated with an increase in the surface integrin β1 and down-regulation of the α4β7 dimer. However, ZFP36L1 is not required for integrin regulation once the ASCs reach the BM (Fig. 6, E and F).

When comparing splenic and BM ASCs at days 14 and 42 after immunization, we observed that CXCR4 is more abundant on splenic CD138^+^NIP^+^IgG1^+^ ASCs prepared at day 42 than on BM ASCs recovered at day 14 (Fig. 6 H and Fig. S5 B), while these two ASC populations are remarkably similar as far as surface expression of the integrins α4 and β1 is concerned (Fig. 6, I and J). Given the prominent role that these molecules are thought to play in guiding an ASC to its survival niche, these observations suggest a possible explanation for the substitution of NP-specific ASCs established in the BM early in the response with the newly immigrated ASCs generated at more advanced stages, the latter being enriched for clones secreting high-affinity antibody. Consistent with the greater control that ZFP36L1 exerts over expression of the integrins α4 and β1 in ASCs formed later in the immune response (Fig. 5, G and H), accumulation of ASCs in the BM of control and Zfp36l1 cKO mice followed different patterns for the high- and low-affinity clones (Fig. S5, G and H). Taken together, these data show that surface expression of the integrin chains α4, β1, and β7 on ASCs undergoes dynamic changes as ASCs migrate from the spleen to the BM. However, their expression on splenic ASCs has to be tightly regulated by ZFP36L1 to facilitate their homing to the BM, especially at the late stages of the primary immune response.

## Discussion

While ZFP36L1 was previously reported to be required for the localization and survival of MZ B cells, it is thought to do so mainly through control of transcription factors such as IRF8 and KLF2, which, when elevated, impose FoB cell–like properties on MZ B cells (Newman et al., 2017). In the present study, ZFP36L1 is shown to directly limit the abundance of GRK2 and of the integrins α4 and β1 in splenic ASCs by binding and restricting levels of their mRNAs. Increased GRK2 results in impaired migration toward S1P and, as a consequence, defective egress of ASCs from the spleen. At the same time, elevated surface expression of integrins α4, β1, and β7 is associated with increased retention of ASCs in the liver and likely contributes to their delayed exit from the spleen. We found that ASC arrival to the BM is accompanied by reduced surface expression of the α4β7 dimer and increased α4 and β1 integrins, presumably reflecting processes leading to eventual lodgement of an ASC in the BM niche. However, as the phenotype of Zfp36l1 cKO mice demonstrates, a premature increase in α4β1 surface abundance and an abnormally high level of α4β7 led to enhanced retention of transitory ASCs in the liver and presumably other sites of VCAM-1, MAdCAM-1, and fibronectin expression.

The integrin dimer α4β1 is thought to be the principal guidance receptor for homing to the BM. However, a role for the α4β7 integrin in this process has also been suggested by transplantation experiments using α4β7-blocking antibody or β7-deficient hematopoietic stem cells (Katayama et al., 2004; Murakami et al., 2016). Furthermore, findings in mice with B cell–specific deletion of the transcriptional factor KLF2 indirectly implicated the α4β7 integrin in ASC migration. In these mice, FoB cells have reduced expression of the α4β7 integrin, and they respond to a T cell–dependent antigen by generating a normal number of splenic ASCs, yet these do not accumulate in the BM (Hart et al., 2011; Winkelmann et al., 2011). While we consistently found an increase in surface expression of the β7 integrin on ZFP36L1-deficient ASCs, we did not detect an increase in KLF2 abundance (data not shown). These observations suggest that in ASCs, KLF2 is not limiting as far as transcription of the β7 integrin is concerned, and other transcriptional factor(s) must be involved.

The reported requirement for strict temporal control of gene expression by ZFP36L1 is in close accord with the mechanism described for enforcement of quiescence by ZFP36L1 and its paralogue ZFP36L2 during the pre–B cell receptor checkpoint (Galloway et al., 2016). In both developmental transitions, demands for a prompt and reversible modulation of gene expression seem to have rendered posttranscriptional regulation superior from an evolutionary perspective to control mediated bytranscriptional factors. The evolutionary advantage for strict control of integrin α4β1 expression by ZFP36L1 comes into sharp relief at later stages of the immune response, when splenic ASCs display increased expression of the α4 and β1 chains compared with ASCs formed early in the GC reaction. Considering the likelihood that mature GCs predominantly produce ASCs secreting high-affinity antibody, the BM homing of such clones should be affected to a greater extent by ZFP36L1 deficiency than the homing of low-affinity clones is. This supposition agrees with our observation of different accumulation patterns for low-affinity and high-affinity ASCs in the BM of control and Zfp36l1 cKO mice.

The elevated expression of CXCR4 and the α4 and β1 integrin chains on ASCs formed late in the response offers a possible explanation as to why such cells might have a competitive edge when it comes to displacement of ASCs already established in the BM niche, a mechanism suggested for affinity maturation in the BM ASC compartment (Radbruch et al., 2006). While at present the only evidence for displacement comes from the appearance of diverse ASCs in the blood of human subjects after vaccination (Odendahl et al., 2005), it is consistent with gradual accumulation and increase in the complexity of V gene mutations in BM ASCs after immunization with NP conjugated to a protein carrier from day 12 onward (Smith et al., 1997; Takahashi et al., 1998). Such a mechanism, however, has been difficult to conceptualize in molecular terms, since high-affinity ASCs lack an obvious means to convert the superior affinity of their receptor to their advantage as the maturation of ASCs leads to a reduction in surface Ig and its associated signal transduction components.

The temporal difference in the surface abundance of CXCR4 and of the α4 and β1 integrins described here circumvents this problem. It also suggests a shift in emphasis in the displacement process from one instructed by B cell receptor–mediated gene expression changes to one of stochastic nature, where probability of the successful event depends on relative levels of the key migratory molecules likely to be defined by maturation status of the GCs. Long-term ASCs of various specificities established in the BM during previously resolved infections are expected to have the highest surface abundance of CXCR4 and of the integrins α4 and β1; accordingly, they are less likely to be substituted by arriving ASCs with fresh specificities generated in the ongoing response. Such a model would also agree with recent work that demonstrated a tendency for the majority of BM long-term plasma cells to be generated late in the response and documented changes in gene expression by early and late GC B cells (Weisel et al., 2016). The study reported that, among other transcripts, CXCR4 mRNA is increased in late GC B cells, a difference that will have to be explored further as it may result from varying proportions of centrocytes and centroblasts in the GCs recovered at different stages of the response. The challenge for the future will be to establish which gene products and signaling pathways drive the difference in the surface phenotype of ASCs formed early and late in the immune response.

## Materials and methods

### Mice, immunization, and in vivo treatment

Mice on the C57BL/6 background were obtained by crosses of mice carrying the following alleles: *Zfp36l1*^*tm1.1Tnr*^ (Hodson et al., 2010), *CD79a*^*tm1(cre)Reth*^ (Hobeika et al., 2006), and *Tg(Fcer2a-cre)5Mbu* (Kwon et al., 2008). Mice carrying the targeted alleles were backcrossed with C57BL/6 mice for at least 10 generations and then intercrossed to generate experimental mice. Mice were bred and maintained in the Babraham Institute Biological Support Unit. Since the opening of this barrier facility in 2009, no primary pathogens or additional agents listed in the Federation of European Laboratory Animal Science Associations recommendations have been confirmed during health monitoring surveys of the stock holding rooms. Ambient temperature was ∼19–21°C, and relative humidity was 52%. Lighting was provided on a 12-h light:12-h dark cycle, including 15-min “dawn” and “dusk” periods of subdued lighting. After weaning, mice were transferred to individually ventilated cages with one to five mice per cage. Mice were fed CRM (P) VP diet (Special Diet Services) ad libitum and received seeds (e.g., sunflower, millet) at the time of cage cleaning as part of their environmental enrichment. All mouse experimentation was approved by the Babraham Institute Animal Welfare and Ethical Review Body. Animal husbandry and experimentation complied with existing European Union and UK Home Office legislation and local standards. All mice used experimentally were between 8 and 12 wk of age and were age and sex matched within experiments; although no sex-associated differences were observed in the results obtained, control mice were sex- and age-matched littermates negative for a Cre-carrying allele.

BrdU (Sigma) was administered at 0.8 mg/ml in drinking water containing 1% sucrose (Thermo Fisher Scientific) the day after reimmunization and for the duration of the secondary response. To label the cells with EdU (Thermo Fisher Scientific), the mice were injected i.p. with 1 mg EdU in 200 µl PBS 2 d after reimmunization. For primary immunization, mice were injected i.p. with 200 µl containing 100 µg NP-KLH (Biosearch Technologies) precipitated in alum (Universal Biologicals). For reimmunization, 50 µg NP-KLH was either precipitated in alum for BrdU- and EdU-labeling experiments or diluted in PBS before injection i.p.

### ELISPOT and ELISA

For ELISPOT, MultiScreen HA mixed cellulose ester plates (Millipore) were coated with NP_23_-BSA (Biosearch Technologies) or NP_2_-BSA (conjugated in-house) in PBS, washed, and blocked with complete medium before applying serially diluted cell suspensions and incubating overnight under 5% CO_2_ at 37°C in IMDM, 10% FCS, 2 mM GlutaMAX, and 50 µM 2-mercaptoethanol. Cells secreting anti-NP antibody were visualized with horseradish peroxidase–conjugated anti–mouse IgG1 antibody (SouthernBiotech) followed by an AEC staining kit (Sigma). The numbers of ASCs were quantified using Immunospot S6 Analyzer (Cellular Technology Limited). ELISA was performed essentially as described previously (Newman et al., 2017).

### Cell preparations and flow cytometry analysis

Preparation of cell suspensions from the spleen and BM and antibody staining for surface markers were performed essentially as described previously (Galloway et al., 2016). Erythrocytes in spleen samples were lysed by incubating cell suspensions for 30 s at room temperature in 1.5 ml ACK buffer (150 mM NH_4_Cl, 10 mM KHCO_3_, and 0.1 mM Na_2_EDTA, pH 7.2–7.4). Lysis was stopped by adding 10 ml of RPMI-1640 (Sigma) and 5% FCS.

A full list of antibodies and other reagents is provided in Table S2. The antibody against GRK2 (OriGene) was labeled using an Alexa Fluor 647 Antibody Labeling Kit from Molecular Probes (Thermo Fisher Scientific) according to the manufacturer’s instructions. To stain for intracellular proteins, cells were first stained for surface markers, and fixable viability dye eFluor780 (eBioscience) was used to identify the cells with an intact plasma membrane. The cells were then fixed in BD Cytofix/Cytoperm (Becton Dickinson) on ice for 30 min, washed in BD Perm/Wash buffer, and either frozen at −80°C in 10% DMSO in FCS or stained overnight at 4°C in BD Perm/Wash buffer containing the corresponding antibody and NIP conjugated to biotin through BSA, followed by washing and staining for 40 min at 4°C with a mix of streptavidin conjugated to PE-Cy7 and anti-mouse IgG1 antibody. For identification of BrdU^+^ cells, the samples were stained for surface markers, then fixed and stained with NIP-biotin and IgG1 antibody followed by fixation and detection of BrdU with the FITC BrdU Flow Kit (Becton Dickinson) according to the manufacturer’s instructions. EdU incorporation was detected with the Click-iT EdU Alexa Fluor 488 Flow Cytometry Assay Kit (Thermo Fisher Scientific). Staining with the antibody against aCasp3^+^ was performed overnight as described above. eFluor780^−^CD4^−^CD8^−^IgD^−^CD138^+^CD267^+^ cells that stained high for intracellular IgG1 but not for NIP were used as a reference population when identifying aCasp3^+^ cells among eFluor780^−^CD4^−^CD8^−^IgD^−^CD138^+^CD267^+^NIP^+^IgG1^+^ cells. The threshold for aCasp3^+^ cells was then verified against a similar threshold for GC B cells (Fig. S3 B). After staining, ∼5 × 10^6^ events were acquired on a Fortessa (Becton Dickinson) flow cytometer, and the data were analyzed using Flowjo software (Becton Dickinson).

To prepare liver lymphocytes, the blood from the liver was flushed by injecting PBS into the hepatic portal vein before collection of the organ. The livers were homogenized in gentleMACS (Miltenyi Biotec) using two cycles of a preset program for spleen. Cell suspensions were loaded onto a discontinuous Percoll gradient (37.5%/72%) and centrifuged at room temperature at 900× *g* for 20 min. The lymphocytes were collected from the interface, washed, and stained for surface markers including NK1.1, TCRβ, CD138, CD267, and CXCR4 as above. Before fixation, one tenth of the cell suspension was taken, washed, mixed with a defined number of AccuCount Beads (Spherotech), and acquired on a flow cytometer. Nine tenths of the cell suspension was fixed and stained for intracellular NIP and IgG1 and analyzed as above. The absolute number of CD138^+^NIP^+^IgG1^+^ cells in the whole sample before fixation was calculated on the basis of counts for TCRβ^+^ cells in the aliquot taken before fixation and in the aliquot that received complete staining.

Blood, taken by cardiac puncture, was collected into tubes containing 300 µl heparin sodium (1,000 U in 1 ml; Wockhardt UK Ltd). The volumes of collected samples were measured, and erythrocytes were lysed by incubating cell suspensions for 30 s in 4 ml ACK buffer twice; lysis was stopped each time by adding 40 ml RPMI 1640 (Sigma), 5% FCS. The cells were stained for surface markers including CD4, TCRβ, CD138, and CD267 as above. The samples were split into two aliquots and processed as described above for the liver samples. The absolute number of CD138^+^NIP^+^IgG1^+^ cells in 1 ml blood was calculated on the basis of counts for CD4^+^TCRβ^+^ cells in the aliquot taken before fixation and in the aliquot that received complete staining.

### Immunostaining of spleen sections

3–4-mm pieces of spleen were fixed in PLP (2% paraformaldehyde, 37.5 mM Na_2_HPO_4_, 75 mM L-lysine, and 10 mM NaIO_4_, pH 7.4) for 4 h at 4°C followed by washes in 30% sucrose in PBS overnight at 4°C. Tissue was frozen in O.C.T. compound on dry ice and sectioned on the cryostat (8 µm thick). Sections were air-dried overnight at room temperature and stored at −20°C. Before staining, the sections were rehydrated in PBS, 0.5% Tween20 and sequentially blocked with PBS, 2% BSA, 2% normal rat serum, and the Streptavidin/Biotin Blocking Kit (Vector Laboratories). Sections were stained in PBS containing 2% BSA, NIP, and antibodies against IgD, CD169 (MOMA-1), and IgG1 at 4°C overnight in a humidified chamber, followed by a 2-h incubation with streptavidin conjugated to Alexa Fluor 555. Slides were washed in PBS, 0.5% Tween20 and mounted in Vectashield Antifade Mounting Medium (Vector Laboratories). Images were acquired at 20× magnification using a Zeiss 780 point-scanning confocal microscope.

### Cell purification and cultures

FoB cells from peripheral and mesenteric LNs were isolated by negative selection. Single-cell suspensions were incubated with biotin-conjugated antibodies against CD5, CD43, Mac, and Ter119 and were then washed and incubated with streptavidin-conjugated magnetic beads (Dynabeads; Thermo Fisher Scientific). Purity of CD19^+^ cells (typically >95%) was confirmed by flow cytometry. For RNA-seq analysis FoB cells were cultured for 48 h in RPMI 1640 medium, 10% FCS, 2 mM GlutaMAX, 1 mM Na-Pyruvate, 50 µM 2-mercaptoethanol containing 10 µg/ml LPS, 10 ng/ml IL4, and 10 ng/ml IL5.

For quantification of GRK2 by Western blotting, FoB cells were purified from spleens essentially as above with the exception of additionally using biotin-conjugated antibodies against CD1d to deplete MZ B cells. Content of MZ B cells after such depletion was comparable between B cell preparations for splenocytes of both genotypes (typically 1–2%). Following culturing of B cells for 96 h with 3.5 µg/ml LPS, 10 ng/ml IL4, and 10 ng/ml IL5, eFluor780^low^CD138^high^B220^low^ cells were sorted using a BD FACSAria III or a BD FACSAria Fusion.

### Western blotting

Sorted CD138^high^B220^low^ cells were washed with PBS and lysed in RIPA buffer (50 mM Tris-HCl, pH 8.0, 150 mM NaCl, 1.0% Igepal CA-630 [NP-40], 0.5% sodium deoxycholate, 0.1% sodium dodecyl sulfate, and 2 mM EDTA) containing protease inhibitors (Sigma) for 10 min on ice. After debris was spun for 12 min at 20,000× *g* at 4°C, the protein content in the lysates was measured with a BCA Protein Assay kit (Pierce). Western blotting of 40 µg of protein for each sample and subsequent signal detection were performed essentially as described previously (Saveliev et al., 2009).

### In vitro migration

Transwell plates (pore size, 5 µm; Costar) were preincubated with chemotaxis buffer (RPMI 1640, 10 mM Hepes, and 1 mM glutaMAX) containing 0.4% BSA at 37°C for 1 h. Plasmablast-like cells obtained by culturing LN FoB cells (see Cell purification and cultures) for 96 h with 3.5 µg/ml LPS, 10 ng/ml IL4, and 10 ng/ml IL5 were washed three times with chemotaxis buffer containing 0.12% BSA and preincubated at 37°C for 50 min. 250 ng/ml CXCL12 (Peprotech) or S1P (Sigma) at 10 nM or 50 nM was added to the lower chamber, whereas plasmablast-like cells were added to the upper chamber (0.8 × 10^6^ cells/well). Cells were allowed to migrate for 3 h at 37°C through the transwell insert. Cells that had migrated into the lower chamber were collected, a defined number of AccuCount Beads (Spherotech) was added to the samples to allow quantification of cell number, and cells were stained with eFluor780 and antibodies against B220, CD138, CD267, and IgG1. The number of eFluor780^−^B220^low^CD138^high^CD267^+^ cells in each sample was quantified by flow cytometry analysis. Efficiency of migration was then expressed as the percentage of cells in the lower chamber as related to the number of identically gated cells in the input population.

### High-throughput sequencing and library preparation

RNA-seq libraries were prepared using an ARTseq Ribosome Profiling Kit (Epicentre; Illumina) according to the manufacturer’s instructions. LPS-activated B cells (see Cell purification and cultures) were treated with 100 µg/ml cycloheximide for 3 min immediately before preparation of cell extracts. 100-bp single-end sequencing of RNA-seq libraries was performed using the HiSeq2500 (Illumina). iCLIP libraries (Galloway et al., 2016) were prepared as described (Vogel et al., 2016).

### Bioinformatic analysis of RNA-seq data

Quality of sequencing data was assessed using FastQC. Reads aligning to ribosomal RNA and Ig loci were filtered out before the analysis. Reads were mapped to mouse genome (GRCm38) using Tophat2 (Kim et al., 2013). Reads aligning to genes were counted using HTSeq-count (Anders et al., 2015), and analysis of differentially expressed genes was performed using the DESeq2 (Love et al., 2014). Cuffnorm (Trapnell et al., 2010) was used to calculate fragments per kilobase of transcript per million mapped reads (FPKM) values. iCLIP sequencing data were visualized using genome assembly GRCm38 with the University of California Santa Cruz (UCSC) genome browser (Kent et al., 2002). GO term–enrichment analysis was performed with gProfiler (Raudvere et al., 2019). A list of genes with average FPKM value across all samples of at least 0.06 and at least one read in each sample was used as a background list of genes. Benjamini-Hochberg false discovery rate was used to correct for multiple testing, and only the terms containing 10–700 genes were considered. Where several significantly enriched terms are hierarchically related, the topmost term is presented in Fig. 4.

### Data availability

The RNA-seq data are available in the Gene Expression Omnibus database under the accession codes GSE146073 (transcriptome of ZFP36L1-sufficient and ZFP36L1-deficient FoB cells) and GSE78249 for the activated B cell iCLIP (Galloway et al., 2016).

### Statistical analysis

The test used and sample sizes are indicated in the figure legends. The Student’s *t* test used was an unpaired two-tailed test. When the variable displayed a very large range, the heterogeneous random errors were stabilized by logarithmic transformation of the data before analysis. All statistical analyses were performed using Prism 8.3.0 software (GraphPad). Statistical tests for data generated by high-throughput sequencing are described in Bioinformatic analysis of RNA-seq data.

### Online supplemental material

Fig. S1 shows representative flow cytometry plots of NP-specific GC B cells and a plot with their frequency, ratio of high-affinity NP antibody to NP antibody with all affinities in sera, serum dilutions required to reach a 50% inhibitory concentration, and enumeration of splenic ASCs. Fig. S2 shows representative flow cytometry plots for newly formed and established CD138^+^NIP^+^IgG1^+^ cells, frequencies of the newly generated CD138^+^NIP^+^IgG1^+^ cells, and representative images of splenic sections from immunized mice. NIP^+^IgG1^+^ cells in the spleens of *Zfp36l1^fl/fl^ CD79a^Cre/+^* mice tend to accumulate normally near venous sinuses and vessel-associated fibers in the red pulp. Fig. S3 shows representative flow cytometry plots used for identification of CD138^+^NIP^+^IgG1^+^ cells in the blood and for identification of aCasp3^+^ cells and a plot with frequencies of aCasp3^+^ CD138^+^NIP^+^IgG1^+^ cells obtained in an independent experiment. The approach used to set up a threshold for quantification of aCasp3^+^ cells among NP-specific ASCs. Fig. S4 shows representative flow cytometry plots for GRK2 signal and a plot with its quantification obtained in an independent experiment, plots with quantification of GRK2 abundance in in vitro–generated plasmablast-like cells and a representative Western blot, abundance of mRNA for the *Itga4*, *Itgb1*, and *Itgb7* genes, a plot with frequencies of α4β7^high^ CD138^+^NIP^+^IgG1^+^ cells, and representative flow cytometry plots for α4 and β1 signals. ZFP36L1-deficient splenic ASCs show an increased surface expression of the integrin chains α4, β1, and β7. Fig. S5 shows a schematic representation of the experiments for analysis of ASCs recovered in responses lasting for 14 and 42 d and for labeling of ASCs newly arrived to the BM, representative flow cytometry plots for CXCR4, α4 and β1 signals and those used for identification of EdU^+^ CD138^+^NIP^+^IgG1^+^ cells, and plots with enumeration of BM ASCs. Table S1 provides the details of differentially expressed genes between LPS-activated B cells from ZFP36L1 cKO animals and control animals. Table S2 lists the antibodies and other reagents used in this study. Different patterns of accumulation in the BM for high- and low-affinity ASCs.

## Supplementary Material

Table S1 provides the details of differentially expressed genes between LPS-activated B cells from ZFP36L1 cKO animals and control animals.

Table S2 lists the antibodies and other reagents used in this study.
