# Supplementary material for: Efficient homing of antibody-secreting cells to the bone marrow requires RNA-binding protein ZFP36L1
Source: J Exp Med. 2020 Dec 11;218(3):e20200504. doi: 10.1084/jem.20200504 (PMC7744253; doi:10.1084/jem.20200504)
Supplement: Table S2 — lists the antibodies and other reagents used in this study. [file JEM_20200504_TableS2.docx]

**Table S2: List of antibodies and other reagents used in the study**

| **Antigen and conjugation** | **Source and Isotype** | **Clone** | **Company** | **Cat. Number** |
| --- | --- | --- | --- | --- |
| β-actin | Mouse IgG1 | AC15 | Sigma | A1978 |
| IgG1-biotin | Goat | polyclonal | Southern Biotech | 1070-08 |
| IgG1-HRP | Goat | polyclonal | Southern Biotech | 1070-05 |
| CD4-BV605 | Rat IgG2a, κ | RM4-5 | BioLegend | 100548 |
| CD8-BV605 | Rat IgG2a, κ | 53-6.7 | BioLegend | 100744 |
| CD19-BUV737 | Rat IgG2a, κ | 1D3 | BD Biosciences | 564296 |
| CD38-PerCP-Cy5.5 | Rat IgG2a, k | 90 | BioLegend | 102721 |
| CD95-BV421 | Armenian Hamster IgG2, λ2 | Jo2 | BD Biosciences | 562633 |
| CD138-BV786 | Rat IgG2a, κ | 281-2 | BD Biosciences | 740880 |
| CD138-PE | Rat IgG2a, κ | 281-2 | BD Biosciences | 553714 |
| CD267-PE | Rat IgG2a, κ | ebio8F10-3 | eBiosciences | 12-5942-81 |
| B220-BUV737 | Rat IgG2a, κ | RA3-6B2 | BD Biosciences | 564449 |
| Bcl6-AF488 | Mouse IgG1, κ | K112-91 | BD Biosciences | 561524 |
| CXCR4-BV421 | Rat IgG2b, κ | 2B11/CXCR4 | BD Biosciences | 562738 |
| GAPDH | Rabbit | D16H11 | Cell Signaling Technology | #5174 |
| IgD-BV605 | Rat IgG2a, κ | 11-26c.2a | BioLegend | 405727 |
| IgG1-BUV395 | Rat IgG1, κ | A85-1 | BD Biosciences | 740234 |
| IgG1-APC | Rat IgG1, κ | X56 | BD Biosciences | 550874 |
| Irf4 eFluor450 | Rat IgG1, κ | 3E4 | eBiosciences | 48-9858-82 |
| Irf4-AF488 | Rat IgG1, κ | 3E4 | BioLegend | 646405 |
| Klf2-APC | Mouse IgG2b | 665333 | R&D Systems | IC5466A-025 |
| NK1.1-BUV395 | Mouse IgG2a, κ | PK136 | BD Biosciences | 564144 |
| TCRβ-PE | Armenian Hamster IgG2, λ1 | H57-597 | BD Biosciences | 553172 |
| aCasp3-AF647 | Rabbit | C92-605 | BD Biosciences | 560626 |
| Grk2 | Rabbit | polyclonal | OriGene | AP21131PU-N |
| Grk2 | Rabbit | polyclonal | Cell Signaling Technology | #3982 |
| α4β7-APC | Rat IgG2a, κ | DATK32 | BioLegend | 120607 |
| CD49d(α4)-APC | Rat IgG2b, κ | R1-2 | BioLegend | 103621 |
| CD29(β1)-AF488 | Armenian Hamster IgG2, λ1 | HM β1-1 | BioLegend | 102211 |
| β7-PE | Rat IgG2a, κ | FIB504 | BioLegend | 321203 |
| β7-FITC | Rat IgG2a, κ | FIB504 | BioLegend | 321213 |
| CD169-FITC | Rat IgG2a | MOMA-1 | Bio-Rad | MCA947G |
| IgD-AF647 | Rat IgG2a, κ | 11-26c.2a | BioLegend | 405708 |
| IgG1-BV421 | Rat IgG | RMG1-1 | BioLegend | 406615 |
| Rabbit IgG-AF647 | Donkey | polyclonal | Jackson ImmunoResearch | 711-605-152 |
| eFluor450  Isotype control | Rat IgG1, κ | eBRG1 | eBiosciences | 48-4301-82 |
| AF488  Isotype control | Rat IgG1, κ | RTK2071 | Biolegend | 400417 |
| AF647  Isotype control | Rabbit IgG1 | DA1E | Cell Signaling | 2985S |
| APC  Isotype control | Mouse IgG2b | 133303 | R&D Systems | IC0041A |
| Mouse IgG- IRDye® 800CW | Goat | polyclonal | Li-Cor | 926-32210 |
| Rabbit IgG- IRDye® 680RD | Goat | polyclonal | Li-Cor | 926-68071 |

| **Reagent** | **Source** | **Cat. Number** | **Application** |
| --- | --- | --- | --- |
| IMDM | Thermo Fisher Scientific | 31980-022 |  |
| RPMI-1640 | Sigma Aldrich | R8758 |  |
| Dimethyl sulfoxide | Sigma Aldrich | D2650 |  |
| Percoll | VWR | GE17-0891-02 |  |
| Dynabeads M-280 Streptavidin | Thermo Fisher Scientific | 11206D |  |
| Heparin sodium | Wockhardt UK Ltd |  |  |
| 4-Hydroxy-3-nitrophenylacetyl-Keyhole Limpet Hemocyanin (NP-KLH) | Biosearch Technologies | N-5060 |  |
| Alu-Gel-S suspension | Universal Biologicals | 12261.01 |  |
| AccuCount Blank Particles | Spherotech | ACBP-50-10 | FC |
| Alexa Fluor 647 Antibody Labeling Kit | Thermo Fisher Scientific | A20186 | FC |
| Fixable Viability Dye eFluor® 780 | eBioscience | 65-0865-14 | FC |
| BD Cytofix/Cytoperm^TM^ | BD Biosciences | 554714 | FC |
| BD Perm/Wash | BD Biosciences |  | FC |
| FITC BrdU Flow Kit | BD Biosciences | 559619 | FC |
| 5-bromo-2-deoxyuridine (BrdU) | Sigma Aldrich | B-5002 | FC |
| 5-ethynyl-2’-deoxyuridine (EdU) | Thermo Fisher Scientific | A10044 | FC |
| 4-Hydroxy-3-iodo-5-nitrophenylacetic acid (NIP)-BSA-biotin | Biosearch Technologies | N-1027 | FC |
| Click-iT EdU Alexa Fluor 488 Flow Cytometry Assay Kit | Thermo Fisher Scientific | C10632 | FC |
| DNase | Thermo Fisher Scientific | AM2238 | FC |
| Streptavidin - PE-Cy7 | Biolegend | 405206 | FC |
| Streptavidin - APC | Biolegend | 405207 | FC |
| OCT compound | Thermo Fisher Scientific | 12678646 | IF |
| Streptavidin/Biotin Blocking Kit | Vector Laboratories | SP-2002 | IF |
| Vectashield Antifade Mounting Medium | Vector Laboratories | H-1000 | IF |
| Streptavidin - Alexa Fluor™ 555 | Thermo Fisher Scientific | S-32355 | IF |
| Maxisorp ELISA plates | Nunc | 439454 | ELISA |
| NP(23)-BSA | Biosearch Technologies | N5050H | ELISA, ELISpot |
| Sigma Fast OPD | Sigma Aldrich | P9187 | ELISA |
| Streptavidin-HRP | Southern Biotech | 7100-05 | ELISA |
| MultiScreenHTS HA Filter Plate | Millipore, Watford, U.K. | MSHAN4510 | ELISpot |
| AEC Staining Kit | Sigma Aldrich | AEC101-1KT | ELISpot |
| CXCL12 | Peprotech | 250-20A | Migration |
| Sphingosine 1-phosphate (S1P) | Sigma Aldrich | 73914 | Migration |
| ARTseq Ribosome Profiling Kit | Epicentre, Illumina | RPHMR12126 | RNA-seq |
